# Determination of the CD148-Interacting Region in Thrombospondin-1

**DOI:** 10.1371/journal.pone.0154916

**Published:** 2016-05-05

**Authors:** Keiko Takahashi, Katherine Sumarriva, Rachel Kim, Rosie Jiang, Dana M. Brantley-Sieders, Jin Chen, Raymond L. Mernaugh, Takamune Takahashi

**Affiliations:** 1 Department of Medicine, Vanderbilt University School of Medicine, Nashville, Tennessee, United States of America; 2 Department of Cancer Biology, Vanderbilt University School of Medicine, Nashville, Tennessee, United States of America; 3 Department of Biochemistry, Vanderbilt University School of Medicine, Nashville, Tennessee, United States of America; Center for Cancer Research, National Cancer Institute, UNITED STATES

## Abstract

CD148 is a transmembrane protein tyrosine phosphatase that is expressed in multiple cell types, including vascular endothelial cells and duct epithelial cells. Previous studies have shown a prominent role of CD148 to reduce growth factor signals and suppress cell proliferation and transformation. Further, we have recently shown that thrombospondin-1 (TSP1) serves as a functionally important ligand for CD148. TSP1 has multiple structural elements and interacts with various cell surface receptors that exhibit differing effects. In order to create the CD148-specific TSP1 fragment, here we investigated the CD148-interacting region in TSP1 using a series of TSP1 fragments and biochemical and biological assays. Our results demonstrate that: 1) CD148 binds to the 1^st^ type 1 repeat in TSP1; 2) Trimeric TSP1 fragments that contain the 1^st^ type repeat inhibit cell proliferation in A431D cells that stably express wild-type CD148 (A431D/CD148wt cells), while they show no effects in A431D cells that lack CD148 or express a catalytically inactive form of CD148. The anti-proliferative effect of the TSP1 fragment in A431D/CD148wt cells was largely abolished by CD148 knockdown and antagonized by the 1^st^, but not the 2^nd^ and 3^rd^, type 1 repeat fragment. Furthermore, the trimeric TSP1 fragments containing the 1^st^ type repeat increased the catalytic activity of CD148 and reduced phospho-tyrosine contents of EGFR and ERK1/2, defined CD148 substrates. These effects were not observed in the TSP1 fragments that lack the 1^st^ type 1 repeat. Last, we demonstrate that the trimeric TSP1 fragment containing the 1^st^ type 1 repeat inhibits endothelial cell proliferation in culture and angiogenesis *in vivo*. These effects were largely abolished by CD148 knockdown or deficiency. Collectively, these findings indicate that the 1^st^ type 1 repeat interacts with CD148, reducing growth factor signals and inhibiting epithelial or endothelial cell proliferation and angiogenesis.

## Introduction

Protein tyrosine phosphatases (PTPs) play important roles in regulating signaling pathways that underlie various cell functions, including cell proliferation, differentiation, and morphogenesis. CD148 (also known as DEP-1, PTPη, or PTPRJ) is a receptor-type PTP characterized by an extracellular region with multiple fibronectin type III-like repeats and a cytoplasmic region with a single catalytic domain [1]. A wide range of cell types express CD148, including epithelial cells, endothelial cells, and hematopoietic cell populations [2–4]. A body of literatures has implicated CD148 in the negative regulation of cell proliferation and transformation. CD148 inhibits endothelial cell proliferation and angiogenesis [5, 6]. CD148 expression is reduced in malignant tumor cells and their transformed phenotype and growth are suppressed when CD148 activity is restored [7–11]. In addition, loss of heterozygosity of CD148 has been described in several types of cancer [12]. Lastly, CD148 reduces growth factor signals by dephosphorylating growth factor receptors and their signaling proteins. These include EGFR [13, 14], HGFR [5, 15], VEGFR2 [16, 17], ERK1/2 [14, 18], PLCγ1 [19], and p85 [20]. Thus, CD148 is thought to function as a suppressor of growth factor signals and inhibit cell growth and transformation. However, the ligand for CD148 has not been identified.

Using a proteomic approach, we recently identified thrombospondin-1 (TSP1) as a CD148-binding extracellular protein and showed that soluble TSP1 binds to CD148 with high affinity and specificity and that its binding increases CD148 catalytic activity and inhibits epithelial or endothelial cell proliferation concomitant with the reduction of growth factor signals (including EGFR, VEGFR2, and ERK1/2) [21]. Thus, TSP1 serves as a functionally important ligand for CD148. TSP1 is a trimeric glycoprotein composed of three identical polypeptide (~145 kDa) chains. The polypeptide chain contains multiple structural elements and interacts with various cell surface receptors that have differing effects, including several integrins, CD36, CD47, and heparan sulfate proteoglycans [22, 23]. Hence, whole TSP1 cannot be used as a specific agonist for CD148. In order to create the CD148-specific TSP1 fragment, here we investigated the CD148-interacting region in TSP1 using a series of TSP1 fragments and biochemical and biological assays. Our results demonstrate that the 1^st^ type 1 repeat in TSP1 interacts with CD148, increases its catalytic activity, reduces growth factor signals, and inhibits epithelial or endothelial cell proliferation and angiogenesis. Thus, the present study further characterized the TSP1-CD148 interaction and offers a new strategy for inhibiting endothelial or epithelial cell growth and angiogenesis.

## Materials and Methods

### Antibodies

The primary antibodies used for immunoblotting and immunoprecipitations: anti-CD148 (clone 143–41), anti-phospho-EGF receptor (Tyr 1173), anti-EGF receptor (1005), anti-CD36 (H-300), anti-β actin (C-2), and anti-γ tubulin (H-183) were from Santa Cruz Biotechnology (Dallas, TX). Anti-phospho-ERK1/2 (Thr202/Tyr204) was from New England BioLabs Inc. (Ipswich, MA). Anti- ERK1/2 was from Upstate Biotechnology Inc. (Lake Placid, NY). Anti-Myc (9E10) was from Vanderbilt Antibody & Protein Resource (Nashville, TN). Anti-phospho-p38 MAP kinase (Thr 180/Tyr182), anti-p38 MAP kinase, and anti-cleaved Caspase-3 were from Cell Signaling Technology, Inc. (Danvers, MA). Secondary antibodies for immunoblotting: HRP-conjugated anti-mouse or anti-rabbit IgG were from GE Healthcare Bio-Sciences (Pittsburgh, PA). Immunofluorescence was performed using anti-von Willebrand Factor (vWF) from Dako North America Inc. (Carpinteria, CA). Alexa Fluor 488-conjugated goat anti-rabbit IgG (Thermo Fisher Scientific, Grand Island, NY) was used as a secondary antibody.

### Reagents

VEGF (human, recombinant) was from R&D Systems Inc. (Minneapolis, MN). Native human TSP1 was from Athens Research & Technology Inc. (Athens, GA). Human IgG, Fc fragment was purchased from Jackson ImmunoResearch Laboratories Inc. (West Grove, PA).

### Plasmids

The human TSP1 cDNA sequences that encode the following amino acid sequences were amplified by PCR from the pGEM2 human TSP1 cDNA (GenBank accession no. X04665.1, Addgene plasmid #12993, Cambridge, MA) [24] and subcloned into HindIII and XbaI sites in APtag-5 vector (GenHunter, Nashville, TN) to add Myc and 6xHis sequences at the C-terminal site; amino-terminus (aa 24–221), procollagen + type 1 repeats (aa 316–547), type 2 repeats (aa 548–690), type 3 repeats and C-terminus (aa 691–1170), oligomerization domain + procollagen + type 1 repeats (aa 261–547), oligomerization domain + procollagen + 1^st^ & 2^nd^ type 1 repeats (aa 261–490), oligomerization domain + procollagen and 1^st^ type 1 repeat (aa 261–429), oligomerization domain + procollagen (aa 261–373), procollagen (aa 316–373), type 1 repeats (aa 379–547), procollagen + 1^st^ type 1 repeat (aa316-429), 2^nd^ & 3^rd^ type 1 repeats (aa 435–547), 1^st^ type 1 repeat (aa 379–429) [The number of amino acid residue includes the signal peptide sequence]. The TSP1 fragment that lacks the 1^st^ type 1 repeat was created as follows: the oligomerization and procollagen sequences (nucleotides 781–1114, aa 261–373) were connected to the 2^nd^ and 3^rd^ type 1 repeats (nucleotides1288-1642, aa 430–547) by inserting a XhoI site and subcloned into the HindIII and XbaI sites in APtag-5 vector. The pCMV-Myc-C expression plasmid for human CD36 was purchased from transOMIC technologies Inc. (Huntsville, AL) and full-length CD36 sequence (nucleotides 66–1481; GenBank accession no. KR710355) was amplified by PCR and subcloned into the BamHI and EcoRI sites in LZRS-IRES-Zeo retroviral vector (a gift from Dr. Al Reynolds, Vanderbilt University) [25]. This subcloning was performed by Custom DNA Constructs (University Heights, OH). The expression plasmids for AP-TSP1 (alkaline phosphatase linked to TSP1) and CD148-Fc (CD148 ectodomain fused to Fc region of human IgG) were described previously [21]. All constructs were confirmed by DNA sequencing.

### Protein Production and Purification

Recombinant human TSP1 fragments were produced and purified as described previously [21]. Briefly, the proteins were produced by HEK293E suspension culture with transient transfection of the plasmids. Culture supernatant was loaded to HisTrap Excel column (GE Healthcare, Pittsburgh, PA), washed with a buffer (20 mM NaH_2_ PO4, 0.5 M NaCl, 20 mM imidazole), and eluted with an elution buffer (20 mM NaH_2_ PO_4_, 0.5 M NaCl, 500 mM imidazole). Proteins were dialyzed against PBS and the purity and quality were assessed by SDS-PAGE and subsequent colloidal blue staining (Thermo Fisher Scientific, Waltham, MA) and immunoblotting using anti-Myc antibody. The proteins were separated in non-reducing as well as in reducing (50 mM DTT) conditions to assess its trimerization. The yields of recombinant proteins were 1.0–28.0 mg/L (mean ± SD; 14.2 ± 8.9 mg/L). AP-TSP1 and CD148-Fc proteins were produced and purified as described previously [21].

### Binding Assay

The purified TSP1 fragments (17 nM) were incubated with either CD148-Fc (44 pmol) or equal mole of Fc fragment of human IgG (control Fc) for 4 h at 4°C. The Fc protein complexes were pulled down using Protein-G sepharose (Sigma-Aldrich, St. Louis, MO) for 1 h at 4°C. The beads were washed with PBS and the bound protein was assessed by anti-Myc immunoblotting. The membrane was reprobed with anti-CD148 antibody to confirm the pull down of CD148-Fc protein. The competition experiment was performed using Reacti-Bind Protein A-coated plates (Thermo Fisher Scientific, Grand Island, NY) as described previously [21]. Briefly, plates were coated with either CD148/Fc (11.3 nM) or equal molar of control Fc. Wells were washed with HBHA buffer (Hank's Buffered Saline Solution containing 0.5 mg/ml BSA and 20 mM Hepes pH 7.0), pre-incubated with TSP1 fragments (25 nM) for 1 h at 4°C, washed, and then incubated with 12 nM of AP-TSP1 with or without 25 nM of TSP1 fragments overnight at 4°C. Wells were washed with HBAH buffer and alkaline phosphatase (AP) activity was assessed using AP assay reagents (GenHunter, Nashville, TN).

### Cell Culture and Stable Cell Preparation

A431D epidermoid cervical carcinoma cells and human renal microvascular endothelial cells (HRMEC) were cultured as described previously [21]. A431D cells stably expressing HA-tagged wild-type (wt) or catalytically inactive mutant (cs) CD148 were prepared as described previously [21]. A431D cells stably expressing CD36 were generated in the same way using the LZRS-IRES-Zeo retroviral vector. Briefly, retrovirus encoding the human CD36 gene was produced using Phoenix packaging cells (provided by Dr. Albert Reynolds, Vanderbilt University) and infected to A431D cells. Stable cells were selected with 400 μg/ml of Zeocin (Thermo Fisher Scientific, Grand Island, NY) and the cells were stained with PE-conjugated anti-CD36 antibody (BD Bioscience, San Jose, CA) and sorted using a BD FACS Aria II flow cytometer (BD Biosciences, San Jose, CA).

### Cell Proliferation Assay

The effects of TSP1 proteins on cell proliferation of A431D and HRMEC cells were assessed as described previously [21]. Briefly, cells were plated in 96-well plates at the density of 2.0 x 10^3^ (A431D) or 1.5 x 10^3^ (HRMEC) cells per well. Serum was reduced to 0.1% FBS overnight (day 0) and then cultured in growth medium supplemented with 2.5% (A431D) or 1.0% (HRMEC) FBS with or without TSP1 proteins at the indicated concentrations. Cell number was assessed at the indicated time points using the Cy-QUANT NF cell proliferation assay kit (Thermo Fisher Scientific, Grand Island, NY) according to the manufacturer’s instruction. The medium was replaced with fresh reagents every 2 days. The concentrations of endogenous TSP1 secreted from the cells were measured using the RayBio Human Thrombospondin-1 ELISA kit (Ray Biotech, Norcross, GA). The levels of secreted TSP1 were lower than 1 nM in both A431D and HRMEC cells in the condition of cell proliferation assay.

### shRNA-mediated CD148 Knockdown

CD148 was knocked down in A431D/CD148wt cells using CD148-targeting shRNA lentiviral particles (Sigma-Aldrich, St. Louis, MO) [21]. Briefly, the cells were plated in 35 mm dishes at 50% density and infected with the shRNA lentivirus [1 x 10^6^ infectious units (IFU)] in medium containing 5 μg/ml Polybrene (Santa Cruz Biotechnology, Dallas, TX). The medium was changed to fresh growth medium 18 h after infection and the cells were used for the proliferation assay. Scrambled shRNA lentivirus (Sigma-Aldrich) was used as a control. CD148 knockdown and its efficiency in HRMECs have been described previously [21].

### Immunoprecipitation and Immunoblotting

Immunoprecipitaion and immunoblotting were performed as described previously [21]. Phosphorylation of EGFR and ERK1/2 was assessed as previously described [21]. Briefly, A431D stable cells were plated in 100-mm dishes at 30% density and then serum was reduced to 2.5% FBS for 12 h. Cells were treated with 12 nM of TSP1 fragments or native TSP1 protein for 15 min and lysed in Nonidet P-40 lysis buffer [20 mM Hepes pH 7.5, 1% NP-40, 150 mM NaCl, 1 mM EDTA, protease inhibitor cocktail (Roche Life Science)]. EGFR was immunoprecipitated from clarified cell lysates (400 μg) using anti-EGFR antibody and immunoblotted with the phospho-specific EGFR antibody. The membrane was reprobed with anti-EGFR antibody. For ERK1/2 phosphorylation, crude lysates (5 μg) were immunoblotted using the phospho-specific ERK1/2 antibody. The membrane was reprobed with anti-ERK1/2 antibody. The downstream signals of CD36 were examined as follows. A431D/CD36 stable cells were plated in 100-mm dishes at 30% density, then serum was reduced to 2.5% FBS for 12 h. Cells were treated with 12 nM of trimeric TSP1 fragments that lacked or contained the 1^st^ type 1 repeat or native TSP1 protein for 18 h. Cells were lysed in Triton lysis buffer [20 mM Tris pH 7.5, 1% Triton X-100, 150 mM NaCl, 2 mM EDTA, protease inhibitor cocktail (Roche Life Science)]. Crude lysates (20 μg) were immunoblotted using the phospho-specific p38 antibody or cleaved Caspase-3 antibody. The membrane was reprobed with anti-p38 or anti-γ-tubulin antibody. Immunoreactions were detected using HRP-conjugated secondary antibodies and visualized using a chemiluminescence detection reagent (Thermo Fisher Scientific, Grand Island, NY).

### PTPase Activity Assay

CD148 phosphatase activity was measured as described previously [21]. Briefly, A431D/CD148wt cells were plated in 100 mm dishes at 30% density, then serum was reduced to 2.5% FBS and cultured for 12 h. Cells were then treated with 12 nM of native human TSP1 protein, TSP1 fragments, or vehicle for 15 min. CD148 was immunoprecipitated from clarified cell lysates (400 μg) using anti-HA affinity matrix (Roche Life Science, Indianapolis, IN) for 4 h at 4°C. The washed immunocomplexes were incubated in the reaction buffer (50 mM sodium acetate, 0.5 mg/ml BSA, 0.5 mM DTT, 5 mM paranitrophenyl phosphate) for 30 min at 30°C, with or without 0.1 mM Na_3_VO_4_. The reaction was stopped with 0.2N NaOH and the amount of cleaved substrate was assessed by measuring OD values at 410 nm. The amount of immunoprecipitated CD148 was assessed by immunoblotting using anti-CD148 antibody. The specificity of the effect was evaluated by treating the cells with the TSP1 fragment in the presence of CD148-Fc (11.3 nM) or equal molar of control Fc.

### Animals

CD148 +/- mouse with C57BL/6 strain (T-736 Ptprj) was purchased from Deltagen Inc. (San Mateo, CA) and genotyped according to the company’s PCR protocol. Wild-type mice obtained from the cross-breeding of heterozygous mice were used as controls.

### Ethics Statement

Mice were housed under pathogen-free conditions and animal experiments were conducted in accordance with AAALAC guidelines and under approval of Institutional Animal Care and Use Committee (IACUC) at Vanderbilt University. Mice were anesthetized with 1–5% isoflurane in oxygen for induction and maintained with 1–3% isoflurane. For the euthanasia method, mice were euthanized by inhalation of carbon dioxide overdose and subsequent cervical dislocation.

### Mouse Sponge Assay

The sponge angiogenesis assay was performed as described previously [26, 27]. Briefly, gel foam sponges (Pharmacia & Upjohn Co, New York, NY) were cut into pieces (2.5–3 mm wide by 5 mm long) and soaked with 100 μl of PBS containing 100 ng of human VEGF (R&D Systems Inc.) plus 100 pmol of a CD148-interacting trimeric TSP1 fragment or vehicle. Two sponges (left and right sides) were implanted into the subcutaneous dorsal frank of 8 week-old wild-type or CD148 knockout mice. Seven days after implantation, mice were injected with 50 μl of 2% tetramethyl rhodamine isothiocyanate (TRITC)-conjugated Dextran (Sigma-Aldrich, St. Louis, MO) PBS solution to label the blood vessels [26, 27]. Five minutes after injection, mice were euthanized and sponges were collected for analysis. Whole-mount sponge images were acquired on an Olympus CK40 inverted microscope (Olympus America Inc. Melville, NY) through an Optronics DEI-750C charge-coupled device video camera (Optronics, Goleta, CA) using CellSens caputure software (Olympus America Inc. Melville, NY). Fluorescence intensity (10x magnification) of TRITC was quantified using ImageJ software (NIH). Data shows the results from six independent sponges in independent mice under each condition. Vessel identity and quantification were confirmed by immunostaining the paraffin sections of sponges for Von Willebrand Factor (vWF), an endothelial cell marker, as described previously [27]. Briefly, 7 μm sections were treated with 0.2 mg/ml Proteinase K (Sigma-Aldrich, St. Louis, MO) in PBS for 5 min at RT. Sections were incubated with anti-vWF antibody overnight at 4°C, followed by Alexa Fluor 488-conjugated goat anti-rabbit IgG, and counterstained with DAPI to visualize nuclei. Microvascular density was quantified in 5 random fields (10x magnification) by scoring fluorescence (vWF) intensity using ImageJ software (NIH).

### Statistical Analysis

Data are expressed as mean ± SEM. Statistical analysis was performed with Prism4 (GraphPad Software Inc., La Jolla, CA). For two-group comparisons, the unpaired Student’s t-test was used to calculate the P value. P < 0.05 was considered as statistically significant.

## Results

### Assessment of the CD148-interacting region in TSP1

**Fig 1:**
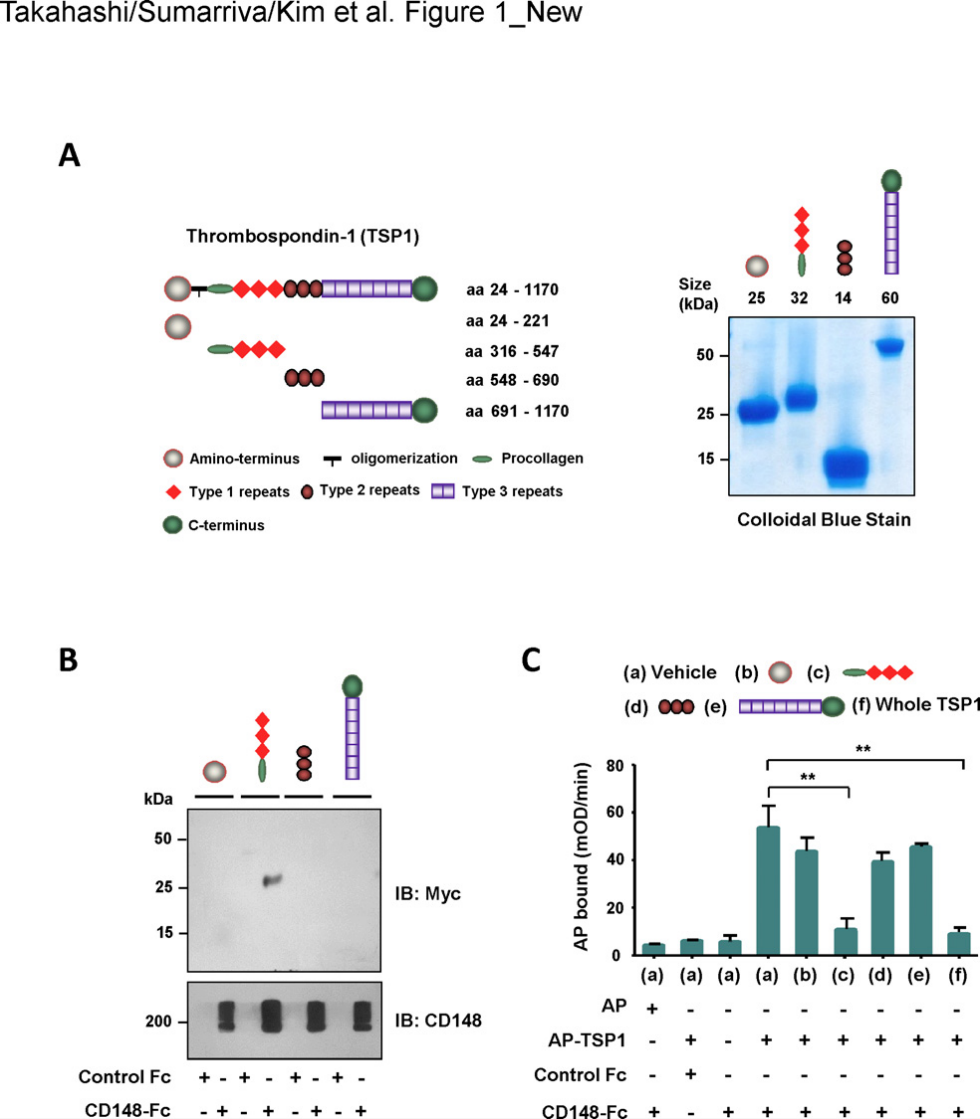
Assessment of CD148-interacting region in TSP1. **(A)** Recombinant TSP1 fragments that correspond to the structural elements were prepared using HEK293E cells. Left panel shows a schematic representation of the TSP1 fragments. The number of amino acid residues includes the signal peptide sequence. Right panel shows colloidal blue stain of the purified TSP1 fragments. Twelve micrograms of protein were separated on a 10% polyacrylamide gel and stained with colloidal blue to assess size and purity. The expected size of protein is also shown. **(B)** TSP1 fragments (17 nM) were incubated with either 44 pmol of CD148-Fc or control Fc (Fc alone). Fc-proteins were pulled down with Protein-G beads and the binding of TSP1 fragments was assessed by immunoblotting using anti-Myc antibody (upper panel). The membrane was reprobed with anti-CD148 antibody to confirm the pull down of CD148-Fc (lower panel). Representative data of five independent experiments is shown. Note: The TSP1 fragment containing the procollagen domain and type 1 repeats binds to CD148-Fc. **(C)** Protein-A plates conjugated with CD148-Fc (11.3 nM) or equal molar of control Fc were incubated with AP-TSP1 or AP (12 nM) in the presence or absence of TSP1 fragments (25 nM) or whole TSP1 protein (25 nM). The bound AP-TSP1 was assessed by an AP activity assay. The data show mean ± SEM of quadruplicate determinations. Representative data of five independent experiments is shown. ** *P* < 0.05 Note: The binding of AP-TSP1 to CD148-Fc is blocked with either a TSP1 fragment containing the procollagen domain and type 1 repeats or whole TSP1 protein.

We have previously reported that soluble TSP1 binds to the ectodomain of CD148 with high affinity and specificity and that TSP1 binding increases CD148 catalytic activity, inhibiting epithelial and endothelial cell proliferation [21]. In order to determine the binding region of TSP1 to CD148, using HEK293E cells, we prepared a series of TSP1 fragments that correspond to its structural elements (**Fig 1A**). The quality of the recombinant proteins was evaluated by SDS-PAGE and subsequent colloidal blue stain and immunoblot analysis. The purified recombinant proteins showed a single band with the expected size in colloidal blue stain and immunoblot analysis (**Fig 1A**, **S1A Fig**). Using these TSP1 fragments, first we conducted a pull-down experiment using CD148-Fc protein in which Fc region of human IgG was fused to the CD148 ectodomain. TSP1 fragments (17 nM) were incubated with CD148-Fc (44 pmol) or equal mole of Fc fragment of human IgG (control Fc) and their binding was assessed by pull-down of Fc proteins and subsequent immunoblotting. Shown in **Fig 1B**, a TSP1 fragment (aa 316–547) that contains the procollagen domain and three type 1 repeats was pulled down with CD148-Fc, while other fragments were not. The bound TSP1 fragment was also detected by the colloidal blue stain as well as by anti-Myc immunoblotting (data not shown). All TSP1 fragments were not pulled down with control Fc. To verify this protein-protein interaction, we next conducted a competition assay using the AP-TSP1 protein [21] in which an alkaline phosphatase was linked to TSP1. Shown in **Fig 1C**, AP-TSP1 (12 nM) bound to CD148-Fc, but not control Fc, and this binding was blocked by the TSP1 fragment (25 nM) containing the procollagen domain and three type 1 repeats as well as by whole TSP1 protein (25 nM). Other TSP1 fragments did not block the binding of AP-TSP1 to CD148-Fc. Similar results were also obtained with higher molar excess (100 nM) of TSP1 fragments (**S2 Fig**). These results suggested that the procollagen domain or type 1 repeats or both interact with the extracellular part of CD148.

### Type 1 repeats are required for TSP1/CD148-mediated inhibition of cell proliferation

**Fig 2:**
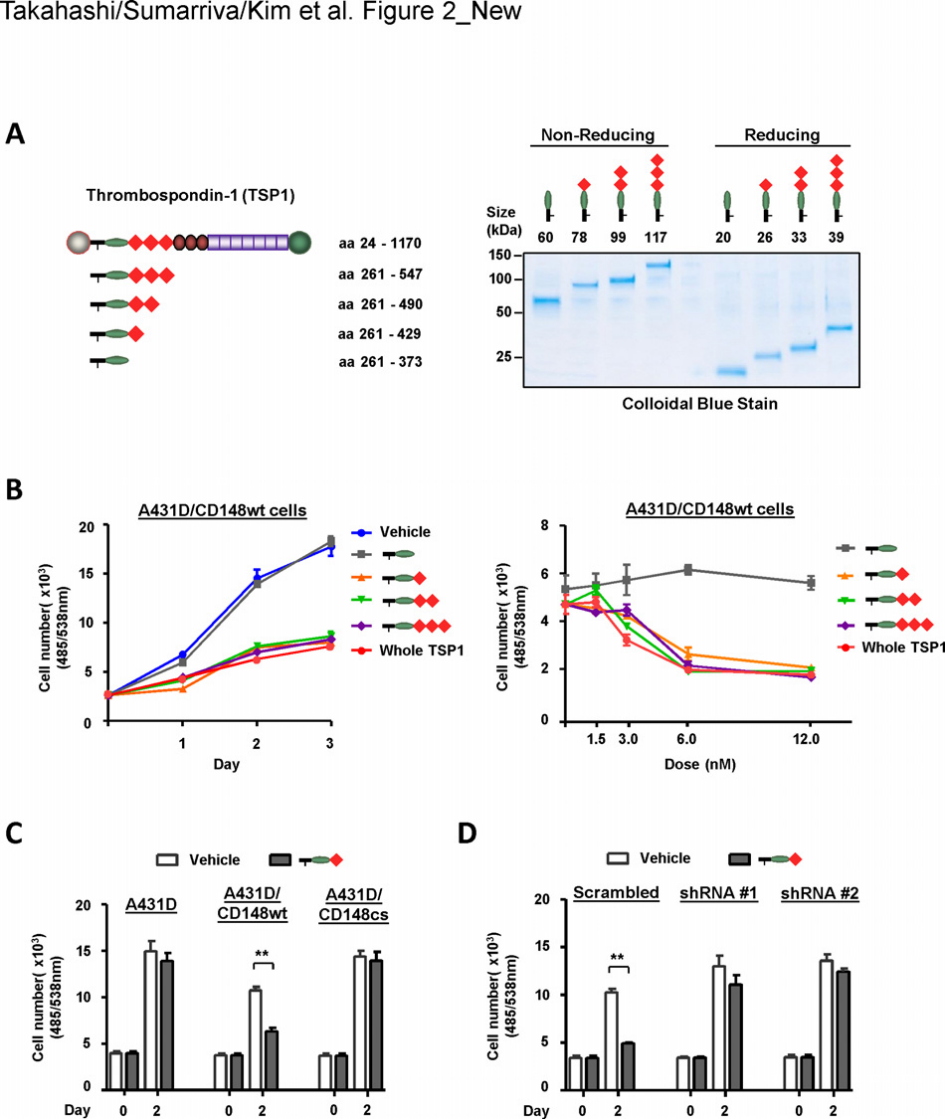
Type 1 repeats are required for TSP1/CD148-mediated cell growth inhibition. **(A)** Trimeric TSP1 fragments containing the procollagen domain and either all three, two, one, or none of type 1 repeats were prepared using HEK293E cells. Left panel shows a schematic representation of the trimeric TSP1 fragments used in this study. The number of amino acid residues includes the signal peptide sequence. Right panel shows colloidal blue stain of the purified TSP1 fragments. Two micrograms of protein were separated on a 4–20% gradient polyacrylamide gel in reducing (+DTT) and non-reducing (-DTT) conditions, then stained with colloidal blue to assess its size, purity, and trimerization. The expected size of protein is also shown. **(B)** A431D cells stably expressing wild-type CD148 (A431D/CD148wt cells) were plated in 96-well plates, starved, and treated with 12 nM of trimeric TSP1 fragments or whole TSP1 protein. Cell density was measured at the indicated time points (left panel). The dose dependency of the effects was also evaluated at day 2 (right panel). The data show mean ± SEM of quadruplicate determinations. Representative data of five independent experiments is shown. **(C)** The effects of a trimeric TSP1 fragment (6.0 nM) containing the procollagen domain and the 1^st^ type 1 repeat on cell proliferation of A431D (lacking CD148) and A431D/CD148cs (stably expressing a catalytically inactive form of CD148) cells are shown. Cell proliferation was assessed as in (B). The data show mean ± SEM of quadruplicate determinations. Representative data of four independent experiments is shown. ** *P* < 0.05 **(D)** CD148 was knocked down in A431D/CD148wt cells using the lentivirus encoding CD148-targeting shRNA (shRNA #1, shRNA #2). The lentivirus encoding scrambled shRNA was used as a control. The cells were subjected to a cell proliferation assay and the effects of CD148 knockdown on growth inhibition of a trimeric TSP1 fragment (12 nM) containing the procollagen domain and the 1^st^ type 1 repeat were assessed. The data show mean ± SEM of quadruplicate determinations. Representative data of four independent experiments is shown. ** *P* < 0.05 Note: CD148 knockdown largely attenuates the TSP1 fragment’s growth inhibitory activity in A431D/CD148wt cells.

We have shown that the interaction of TSP1 with CD148 inhibits cell proliferation in A431D cells when CD148 is introduced [21]. Using this system, we next asked if the TSP1 fragment containing the procollagen domain and type 1 repeats exhibits the same biological effects in A431D cells. Further, we created TSP1 fragments containing the procollagen domain and either all three, two, one, or none of the type 1 repeats to narrow down the CD148-interacting region in TSP1 with this biological assay (**Fig 2A**). We and others have demonstrated that ectodomain-mediated oligomerization is a potential mechanism of CD148 activation using the antibodies raised against the ectodomain sequence of CD148 [5, 28, 29]. Indeed, TSP1 is a trimeric protein [30]. Therefore, we created the trimeric TSP1 fragments for this biological study by adding the oligomerization sequence (~ 50 residues) [31] of TSP1 to the fragments (**Fig 2A**). The quality of the recombinant proteins was evaluated by SDS-PAGE and subsequent colloidal blue stain and immunoblot analysis (**Fig 2A**, **S1B Fig**). Trimerization of the TSP1 fragments was assessed by running the proteins in non-reducing conditions. Shown in **Fig 2A** and **S1B Fig,** the purified recombinant proteins showed a single band with the expected size in colloidal blue stain and immunoblot analysis and its trimerization was confirmed in non-reducing conditions. Subsequently, the biological activity of these fragments was assessed using the A431D cells that stably express wild-type (wt) CD148 at physiological levels but lack the expression of CD36 and CD47, known growth inhibitory TSP1 receptors (hereafter A431D/CD148wt cells) [21]. The cells were treated with biologically relevant concentrations (<100 nM) of TSP1 fragments [32]. Shown in **Fig 2B**, the trimeric TSP1 fragments containing the procollagen domain and type 1 repeats dose-dependently inhibited the cell proliferation of A431D/CD148wt cells at levels comparable to those seen with whole TSP1, while a trimeric TSP1 fragment containing the procollagen domain alone showed no effects. In addition, the trimeric TSP1 fragment that contains the amino-terminus domain showed no effects (data not shown). Of note, the 1^st^ type 1 repeat was sufficient to inhibit cell proliferation of A431D/CD148wt cells. The specificity of this effect was further evaluated using A431D cells (which lack CD148 expression) and A431D/CD148cs cells (which stably express a catalytically inactive form of CD148), as well as by CD148 knockdown, as described previously [21]. CD148 was knocked down using the lentivirus encoding CD148-targeting shRNA. Downregulation (80–90%) of CD148 was confirmed by immunoblot analysis (**S3 Fig**). Shown in **Fig 2C,** a TSP1 fragment (procollagen domain + 1^st^ type 1 repeat) that inhibited cell proliferation of A431D/CD148wt cells showed no effects in A431D and A431D/CD148cs cells. Furthermore, the growth inhibitory activity of this fragment in A431D/CD148wt cells was largely diminished by CD148 knockdown, while the fragment inhibited cell proliferation of A431D/CD148wt cells treated with scrambled (control) shRNA (**Fig 2D**). These results indicated that type 1 repeats are required for TSP1/CD148-mediated cell growth inhibition and that the 1^st^ type 1 repeat is sufficient for this effect. It is of note that a monomeric form of the TSP1 fragment lacks this growth inhibitory activity (**S4 Fig**). Shown in **S5A Fig**, a monomeric form of the TSP1 fragment competed less with the binding of AP-TSP1 to CD148-Fc when compared with its trimeric form fragment based on molar concentrations. Since three-fold molar excess of the monomeric fragment effectively blocked the binding of AP-TSP1 to CD148-Fc as its trimeric fragment (**S5B Fig)**, this may be related to the valency of the CD148 binding site in the fragment. It is of importance that a monomeric fragment did not inhibit cell proliferation of A431D/CD148wt cells even when three-fold molar excess of fragment was added (**S4 Fig**), indicating lack of activity of this fragment in inhibiting proliferation of A431D/CD148wt cells. The accumulated CD148 distribution observed in the trimeric fragment-treated cells also suggests that oligomerization of CD148 plays a key role in TSP1-mediated CD148 activation (**S7B Fig**).

### The 1^st^ type 1 repeat is required for TSP1/CD148-mediated inhibition of cell proliferation

**Fig 3:**
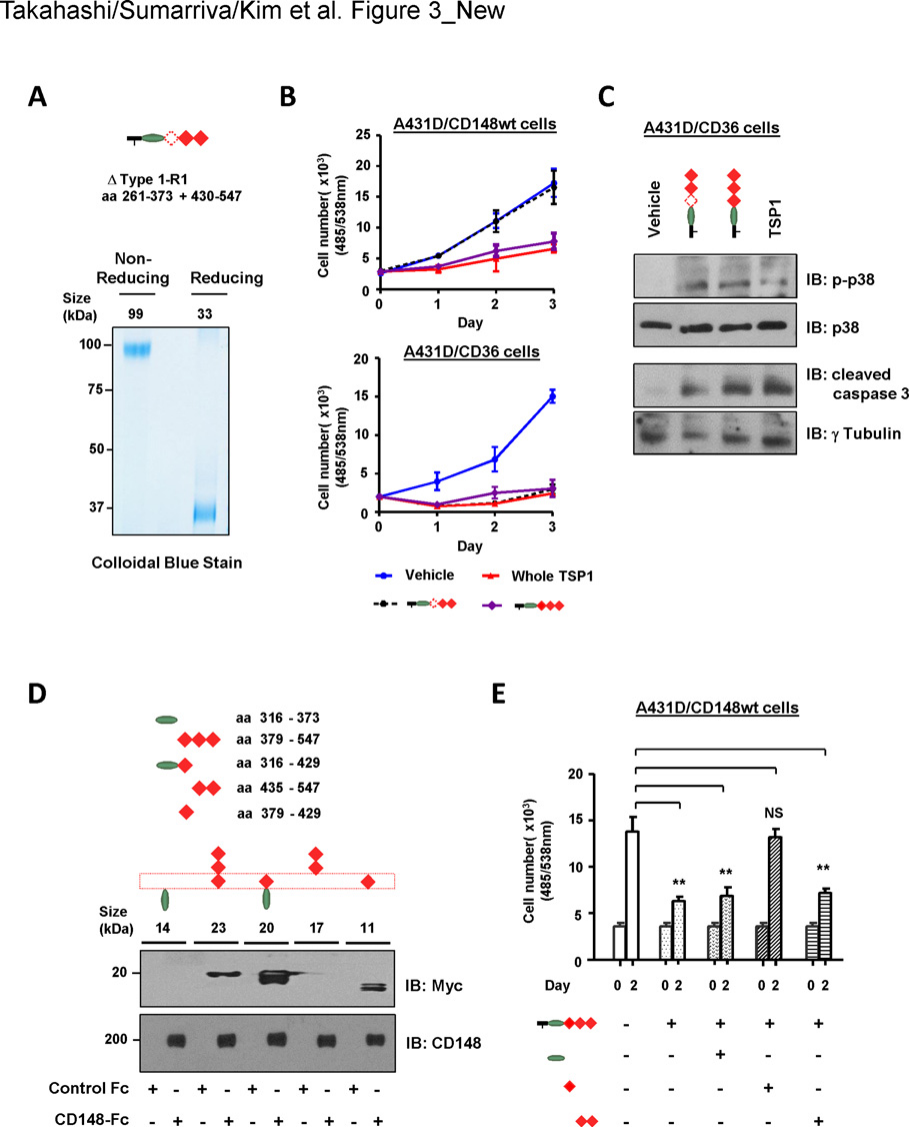
The 1^st^ type 1 repeat is required for TSP1/CD148-mediated cell growth inhibition. **(A)** The trimeric TSP1 fragment (ΔType1-R1) that contains the procollagen domain and the 2^nd^ and 3^rd^, but not the 1^st^, type 1 repeat was prepared using HEK293E cells. Upper panel shows a schematic representation of the trimeric TSP1 fragment that lacks the 1^st^ type 1 repeat. Amino acid residues (aa 374–429) of the 1^st^ type 1 repeat were deleted. Lower panel shows colloidal blue staining of the purified ΔType1-R1 fragment. Five micrograms of protein were separated on a 10% polyacrylamide gel in reducing (+DTT) and non-reducing (-DTT) conditions and stained with colloidal blue to assess size, purity, and trimerization. The expected size of protein is also shown. **(B)** A431D/CD148wt or A431D/CD36 (stably expressing CD36) cells were treated with 12 nM of trimeric TSP1 fragments that lack or contain the 1^st^ type 1 repeat or whole TSP1 protein. Cell density was measured at the indicated time points. The data show mean ± SEM of quadruplicate determinations. Representative data of four independent experiments is shown. Note: The ΔType1-R1 fragment shows no growth inhibitory activity in A431D/CD148wt cells, while it inhibits cell proliferation in A431D/CD36 cells. **(C)** A431D/CD36 cells were treated with trimeric TSP1 fragments (12 nM) that lacked or contained the 1^st^ type 1 repeat or whole TSP1 protein (12 nM) for 18 h. Tyrosine phosphorylation of p38 and cleaved caspase 3 was assessed by immunoblotting using the phopho-specific p38 (pThr180+Tyr182) or cleaved caspase 3 antibodies. The membranes were reprobed with antibodies to total p38 or γ-tubulin. Representative data of four independent experiments is shown. **(D)** A series of monomeric TSP1 fragments were prepared from the regions containing the procollagen domain and type 1 repeats as shown in a schema on right side. Each fragment (17 nM) was incubated with either 44 pmol of CD148-Fc or control Fc (Fc alone), and Fc-proteins were pulled down with protein-G beads. Bound TSP1 fragments were assessed by anti-Myc immunoblotting (upper panel). Half of each sample was subjected to anti-CD148 immunoblotting to confirm the pull down of CD148-Fc (lower panel). Representative data of five independent experiments is shown. Note: TSP1 fragments that contain the 1^st^ type 1 repeat bind to CD148-Fc. **(E)** A431D/CD148wt cells were treated with or without indicated TSP1 fragments (36 nM) for 1 h, then a trimeric TSP1 fragment (12nM) containing the procollagen domain and type 1 repeats was added to the medium. Cell proliferation was assessed at day 2. The data show mean ± SEM of quadruplicate determinations. Representative data of five independent experiments is shown. ** *P* < 0.05 Note: Only the 1^st^ type 1 repeat blocks cell growth inhibition induced by the trimeric TSP1 fragment.

Since the 1^st^ type 1 repeat was sufficient for TSP1/CD148 cell growth inhibition, we next asked if the 1^st^ type 1 repeat is the responsible region for mediating TSP1/CD148 inhibition of cell growth. To address this issue, we created a trimeric TSP1 fragment (ΔType1-R1) that contains the procollagen domain and the 2^nd^ and 3^rd^ type 1 repeats but lacks the 1^st^ type 1 repeat (aa 374–429) (**Fig 3A**). The quality of the recombinant protein was confirmed by SDS-PAGE and subsequent colloidal blue stain and immunoblot analysis (**Fig 3A**, **S1B Fig)**. The biological activity of the 1^st^ type 1 repeat-deleted TSP1 fragment was assessed in A431D/CD148wt cells. Shown in **Fig 3B,** the ΔType1-R1 fragment showed no growth inhibitory activity in A431D/CD148wt cells, while the trimeric TSP1 fragment that contains all three type 1 repeats or whole TSP1 protein inhibited its cell growth. It is well known that the 2^nd^ and possibly the 3^rd^ type 1 repeats bind to CD36 and inhibit cell proliferation [33, 34]. Since the deletion of the 1^st^ type 1 repeat may change the structure of the trimeric TSP1 fragment and abolish its biological activity, we also examined whether the ΔType1-R1 fragment has the ability to activate CD36. The A431D cells that stably express CD36 (A431D/CD36 cells) were prepared (**S6 Fig**) and used to address this issue. Shown in **Fig 3B** (right panel), the 1^st^ type 1 repeat-deleted as well as -undeleted trimeric TSP1 fragment inhibited the cell proliferation of A431D/CD36 cells to the same degree as whole TSP1. Furthermore, these TSP1 fragments activated the p38 and caspase-3 pathways, the reported downstream pathways of CD36 [35] (**Fig 3C**). Collectively, these findings indicate that the 1^st^ type 1 repeat is responsible for mediating TSP1/CD148 inhibition of cell proliferation. This was further confirmed by the CD148-Fc pull-down experiment. Shown in **Fig 3D**, a series of TSP1 fragments (monomeric) were prepared for the region of procollagen domain and type 1 repeats. The quality of the recombinant proteins was confirmed by immunoblot analysis (**S1C Fig).** These TSP1 fragments (17 nM) were incubated with CD148-Fc (44 p mol) or equal mole of control Fc and its binding was assessed by pull-down of Fc proteins and subsequent immunoblotting. Shown in **Fig 3D**, the TSP1 fragments that contain the 1^st^ type 1 repeat were pulled down with CD148-Fc, while the fragments that lack this region were not. Control Fc (Fc alone) was unable to pull down the fragments. Last, we asked if the fragment of the 1^st^ type repeat antagonizes the activity of a trimeric TSP1 fragment in inhibiting cell proliferation of A431D/CD148wt cells. Shown in **Fig 3E**, three-fold molar excess of the 1^st^, but not the 2^nd^ and 3^rd^, type 1 repeat fragment largely antagonized the activity of a trimeric TSP1 fragment (containing the procollagen domain and type 1 repeats) in inhibiting proliferation of A431D/CD148wt cells. The fragment of procollagen domain showed no effects. Taken together, these results indicated that the 1^st^ type 1 repeat is responsible for the TSP1/CD148-mediated inhibition of cell proliferation.

### Trimeric TSP1 fragments that contain the 1^st^ type 1 repeat increase CD148 catalytic activity

**Fig 4:**
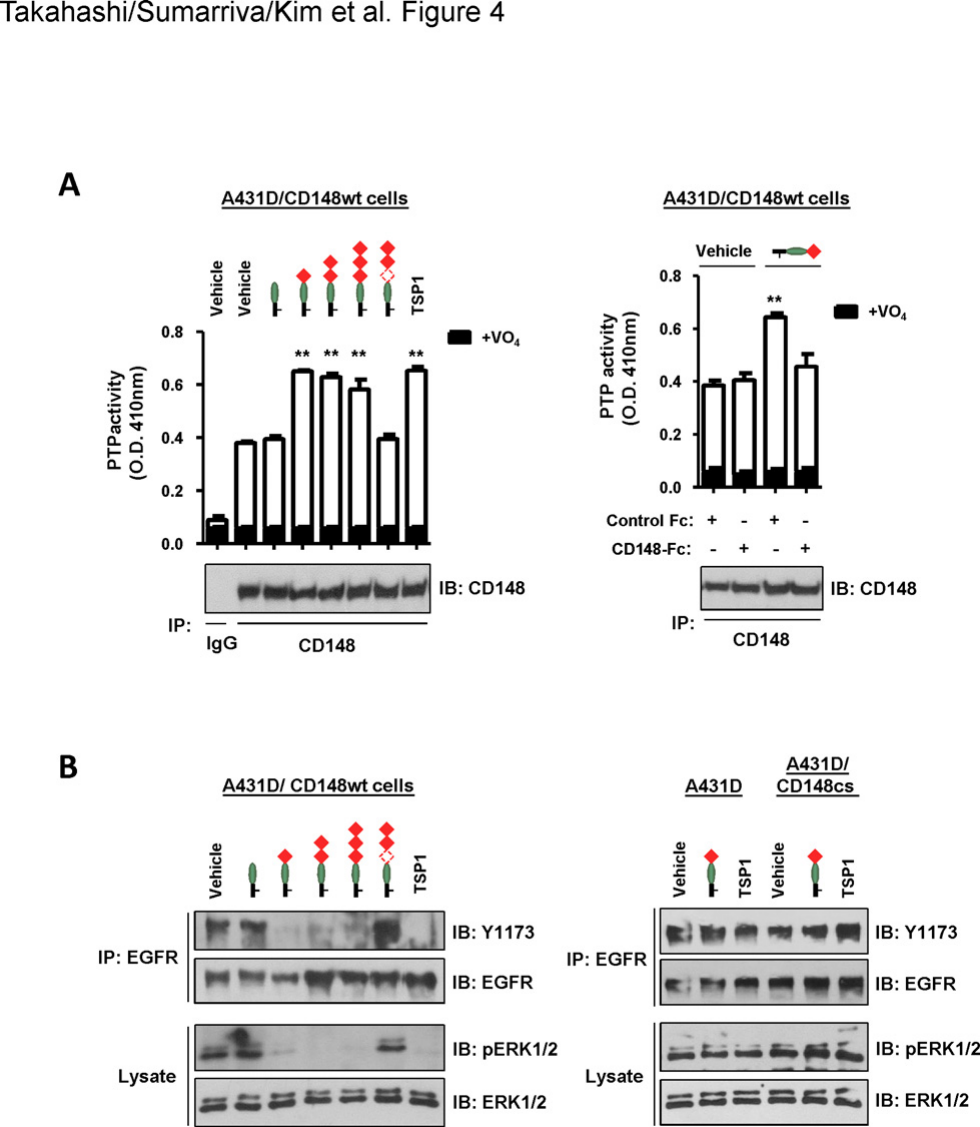
Trimeric TSP1 fragments that contain the 1^st^ type 1 repeat increase CD148 catalytic activity and reduce tyrosine phosphorylation of EGFR and ERK1/2 in A431D/CD148wt cells. **(A)** Left: A431D/CD148wt cells were treated with the indicated trimeric TSP1 fragments (12 nM) or whole TSP1 protein (12 nM) for 15 min. CD148 was immunoprecipitated using anti-CD148 antibody or class-matched control IgG. The washed immunocomplexes were subjected to a PTP activity assay with or without 1 mM sodium orthovanadate (VO_4_). The amount of CD148 in the immunocomplexes was evaluated by immunoblotting using anti-CD148 antibody (lower panel). The data show mean ± SEM of quadruplicate determinations. Representative data of five independent experiments is shown. ** *P* < 0.05 vs. vehicle-treated cells. Right: To assess the specificity of the effect, a trimeric TSP1 fragment containing the procollagen domain and the 1^st^ type 1 repeat was added to A431D/CD148wt cells with 11.3 nM of CD148-Fc or control Fc (Fc alone), then CD148 catalytic activity was assessed as in left panel. The data show mean ± SEM of quadruplicate determinations. Representative data of five independent experiments is shown. ** *P* < 0.05 vs. vehicle-treated cells. Note: CD148-Fc, but not control Fc, abolishes the activity of the TSP1 fragment to increase CD148 catalytic activity. **(B)** Left: A431D/CD148wt cells were treated with the indicated trimeric TSP1 fragments (12 nM) or whole TSP1 protein (12 nM) for 15 min. Tyrosine phosphorylation of EGFR (immunoprecipitated) and ERK1/2 was assessed by immunoblotting using the phopho-specific EGFR (Y1173) or ERK1/2 (T202/Y204) antibodies. The membranes were reprobed with antibodies to total EGFR or ERK1/2. Representative data of four independent experiments is shown. Right: A431D and A431D/CD148cs cells were treated with a trimeric TSP1 fragment (12 nM) that contains the procollagen domain and the 1^st^ type 1 repeat or whole TSP1 protein (12 nM), and tyrosine phosphorylation of EGFR (immunoprecipitated) and ERK1/2 was assessed as in left panel. Representative data of four independent experiments is shown. Note: No effects are observed in A431D and A431D/CD148cs cells.

We next asked if the trimeric TSP1 fragment that contains the 1^st^ type 1 repeat increases the catalytic activity of CD148. A431D/CD148wt cells were treated with a series of the trimeric TSP1 fragments (12 nM) and the catalytic activity of CD148 was assessed by the measurement of PTP activity of immunoprecipitable CD148 and by dephosphorylation of its substrates (EGFR, ERK1/2), as described previously [21]. Shown in **Fig 4A** (left panel), the trimeric TSP1 fragments that contain the procollagen domain and either all three, two (1^st^ and 2^nd^), or one (1^st^) type 1 repeats and whole TSP1 significantly increased the catalytic activity of CD148 in A431D/CD148wt cells, while the trimeric TSP1 fragment that contains the procollagen domain alone or lacks the 1^st^ type 1 repeat did not. No difference was observed in the amount of immunoprecipitable CD148 between vehicle- and TSP1 fragments or TSP1-treated cells (lower panel). The specificity of this effect was evaluated using CD148-Fc. The right panel shows the result of the trimeric TSP1 fragment that contains the procollagen domain and the 1^st^ type 1 repeat. The effect of this fragment to increase CD148 catalytic activity was antagonized by CD148-Fc (11.3 nM), but not equal molar of control Fc (Fc alone). Consistent with these results, the trimeric TSP1 fragments that increased CD148 catalytic activity, as well as whole TSP1, reduced the phosphorylation levels of EGFR and ERK1/2, defined CD148 substrates [13, 14] in A431D/CD148wt cells, while the trimeric TSP1 fragment that contains the procollagen domain alone or lacks the 1^st^ type 1 repeat did not (**Fig 4B, left panel**). The right panel in **Fig 4B** shows the effects of the trimeric TSP1 fragment that contains the procollagen domain and the 1^st^ type 1 repeat in A431D or A431D/CD148cs cells. Shown in this panel, this effect was not observed in these A431D cells that lack CD148 activity. Collectively, these findings indicated that the 1^st^ type 1 repeat increases CD148 catalytic activity and results in dephosphorylation of its substrates in intact cells. It is of note that these activities were not observed in a monomeric form of TSP1 fragment (**S7A Fig**). It was shown that EGFR phosphorylation is increased in A431 cells by TSP1 treatment (214 nM, 30 min) [36]. This finding is contradictory to our results. Given the facts that the same TSP1 treatment (214 nM, 30 min) also reduces phosphorylation of EGFR and ERK1/2 in A431D/CD148wt cells and that CD148 expression is much lower in A431 cells as compared with A431D/CD148wt and HRMEC cells (**S8 Fig**), it is likely that a low level of CD148 expression caused different effects of TSP1 in A431 cells.

### A trimeric TSP1 fragment that contains the 1^st^ type 1 repeat inhibits endothelial cell proliferation and angiogenesis via CD148

**Fig 5:**
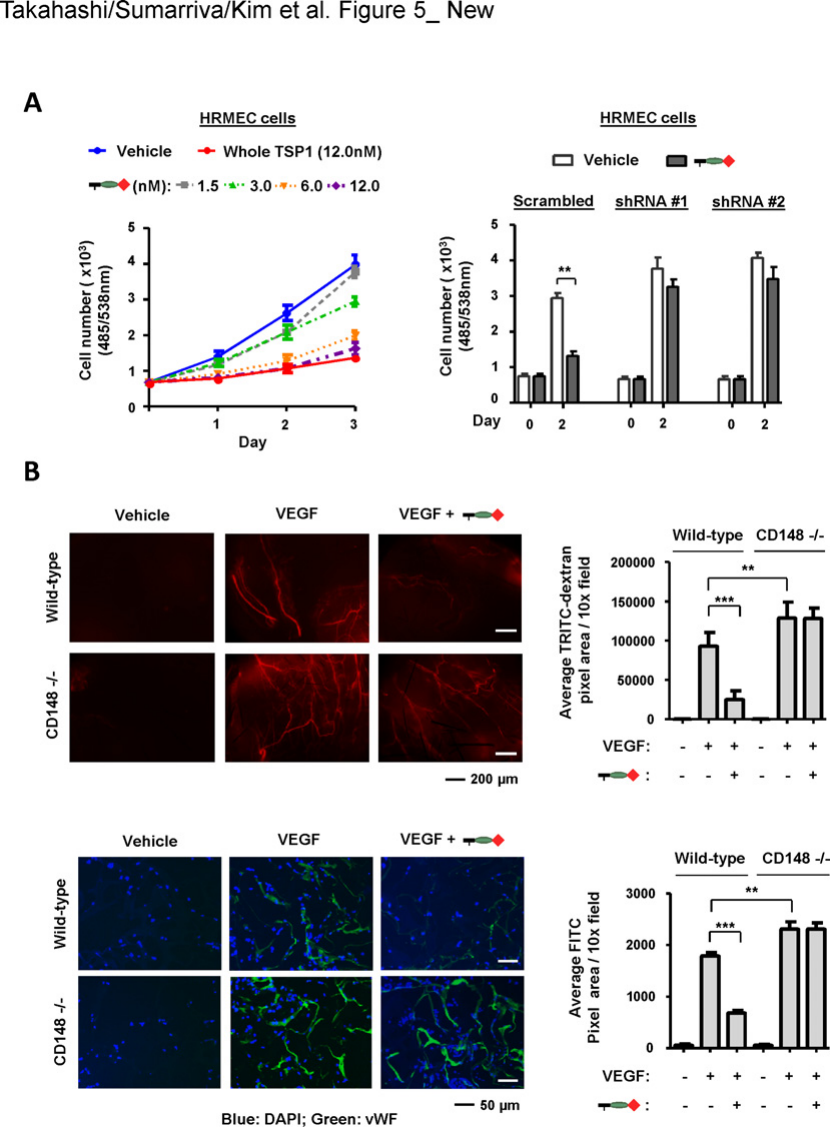
A CD148-interacting trimeric TSP1 fragment inhibits endothelial cell proliferation and angiogenesis. **(A)** HRMEC cells were treated with the trimeric TSP1 fragment (1.5, 3.0, 6.0, 12.0 nM) containing the procollagen domain and the 1^st^ type 1 repeat or whole TSP1 (12 nM) and its effects on cell proliferation were assessed (left panel). The effects of CD148 knockdown were also assessed with 12 nM of the TSP1 fragment (right panel). The data show mean ± SEM of quadruplicate determinations. Representative data of five independent experiments is shown. ** *P* < 0.05 Note: CD148 knockdown largely attenuates the activity of the TSP1 fragment to inhibit cell proliferation of HRMEC cells. **(B)** Upper panels: Gelfoam sponges loaded with vehicle or 100 ng VEGF plus or minus 100 pmol of the trimeric TSP1 fragment containing the procollagen domain and the 1^st^ type 1 repeat were subcutaneously implanted into the dorsal flank of either wild-type or CD148 knockout mice. At day 7, the mice were injected intravenously with 2% TRITC-dextran to label vessels, then the sponges were excised for analysis. Left panels show representative results of whole sponges under a fluorescence microscope. Right panel shows the TRITC-based quantification of vessel density in whole sponges. TRITC-positive pixel area was measured. Data show mean ± SEM of six sponges from independent mice. ** *P* < 0.05, *** *P* < 0.01 Note: A CD148-interacting trimeric TSP1 fragment inhibits VEGF-induced angiogenesis in wild-type, but not CD148 knockout, mice. Lower panels: Paraffin sections were processed from each sponge. Vessel density was assessed by vWF immunostaining. The sections were counterstained with DAPI. Left panels show representative results of vWF immunostaining in each condition. Right panel shows FITC-based quantification of vessel density in each group. Data show mean ± SEM of six sponges from independent mice. ** *P* < 0.05, *** *P* < 0.01

We and others have shown that CD148 is expressed in endothelial cells and negatively regulates endothelial cell proliferation and angiogenesis [5, 6]. Therefore, we next asked if the trimeric TSP1 fragment that interacts with CD148 exhibits this activity. The 2^nd^ and possibly the 3^rd^ type 1 repeats are known to interact with CD36 and inhibit endothelial cell proliferation [33, 34]. To eliminate the effects of CD36-mediated angiogenesis inhibition, the trimeric TSP1 fragment that contains the procollagen domain and the 1^st^ type 1 repeat was used for this study. Shown in **Fig 5A** (left panel), the CD148-interacting trimeric TSP1 fragment dose-dependently inhibited cell proliferation of human renal microvascular endothelial cells (HRMEC) [21] that express CD148, and this effect was largely abolished by lentivirus-mediated CD148 knockdown (right panel). The efficiency of CD148 knockdown was greater than 80% as described previously [21]. Interestingly, this TSP1 fragment inhibited cell proliferation of HRMEC at the level of whole TSP1, though whole TSP1 inhibits endothelial cell proliferation through CD36 as well as CD148. This may be due to the low level expression of CD36 in these endothelial cells [21] or sufficient activity of CD148 to inhibit endothelial cell proliferation. It is of note that this fragment reduced the tyrosine phosphorylation of VEGFR2 and ERK1/2 upon VEGF stimulation (**S9 Fig**). In addition, we noted that the trimeric TSP1 fragment containing the procollagen domain alone showed no effects in HRMEC cells (data not shown). Based on these results, we next asked if this TSP1 fragment inhibits angiogenesis *in vivo*. This issue was addressed by the sponge angiogenesis assay, where VEGF (100 ng) was used as an inducer. Angiogenesis was evaluated by TRITC-dextran perfusion as well as by vWF immunofluorescense staining. Shown in **S10 Fig**, CD148 was expressed in angiogenic vessels in the sponge, which were induced by VEGF, and the trimeric TSP1 fragment (100 pmol) containing the procollagen domain and the 1^st^ type 1 repeat significantly inhibited angiogenesis (**Fig 5B**). Furthermore, this effect was abolished by CD148 deficiency (**Fig 5B**). Of interest, CD148 knockout mice showed significantly increased VEGF angiogenesis as compared with wild-type mice, indicating a role of CD148 in negative regulation of VEGF angiogenesis. It is of note that the trimeric TSP1 fragment that contains the procollagen domain alone did not inhibit angiogenesis in wild-type mice (**S11 Fig**). Collectively, these results indicated that the trimeric TSP1 fragment that contains the 1^st^ type 1 repeat inhibits endothelial cell proliferation and angiogenesis through CD148.

## Discussion

The present study examined the CD148-interacting region in TSP1 using biochemical and biological assays. Our data demonstrates that the 1^st^ type 1 repeat of TSP1 interacts with CD148, increasing its catalytic activity and resulting in tyrosine dephosphorylation of defined substrates, and inhibiting cell proliferation. Our results also indicate that the CD148-interacting TSP1 fragment inhibits proliferation of A431D cells, an epidermoid cervical carcinoma cell line, when CD148 is expressed. The finding suggests that this fragment may be used for anti-cancer therapy of epithelial tumors that express CD148 as well as for angiogenesis inhibition.

TSP1 has been shown to inhibit endothelial cell proliferation, migration, and angiogenesis [23, 37, 38]. Subsequent studies have shown that the main anti-angiogenic sequences reside within the type 1 repeats [39, 40]. Tolsma *et al*. created a series of TSP1 fragments by chymotrypsin digestion of native TSP1 and with a baculovirus expression system and showed that the majority of the anti-angiogenic activity of TSP1 resides in the region containing the procollagen domain and type 1 repeats [39]. Furthermore, the authors demonstrated that peptides from the 2^nd^ and 3^rd^, but not the 1^st^, type 1 repeats inhibit corneal angiogenesis. Iruela-Arispe *et al*. reported similar results [40]. In this study, the authors created a series of GST-TSP1 fragments through prokaryotic expression system and demonstrated that the 2^nd^ and 3^rd^, but not the 1^st^, type 1 repeats inhibit chorioallantoic membrane (CAM) angiogenesis induced by VEGF and bFGF [40]. They also showed that peptides derived from these regions inhibit CAM angiogenesis. Furthermore, CD36 was shown to interact with the 2^nd^ and 3^rd^ type 1 repeats, induce endothelial cell apoptosis, and largely account for the anti-angiogenic activity of these regions [33–35, 41, 42]. Thus, although CD148 interacts with the 1^st^ type 1 repeat and inhibits endothelial cell growth and angiogenesis, previous studies have failed to detect anti-angiogenic activity in the 1^st^ type 1 repeat sequence. However, some differences exist between these previous studies and our present work. First, previous studies utilized monomeric TSP1 peptides or dimeric GST-fusion recombinant proteins [43] to map the anti-angiogenic region of TSP1, while our TSP1 fragment is a trimeric form. Given the fact that a monomeric form of TSP1 fragment is inactive in increasing CD148 activity in A431D/CD148wt cells, proper oligomerization of the TSP1 fragment may be critical to activate CD148. Indeed, Tolsma *et al*. also showed that angiogenesis is strongly inhibited by the trimeric TSP1 fragment that contains the procollagen domain and 1^st^ type 1 repeat [39]. Second, the TSP1 fragments or peptides used in the previous studies were non-glycosylated, while our TSP1 fragments were produced with mammalian cells; thereby, they could be glycosylated. Indeed, we examined the glycosylation of a minimally active TSP1 fragment by mass spectrometry and observed the reported glycosylation (C-linked glycosylation on Trp and O-linked glycosylation on Ser) [44] in this fragment. Furthermore, the GST-TSP1 fragment prepared by a prokaryotic expression system may not be folded accurately. Thus, the form and nature of our recombinant proteins differ from those in previous studies. This may be the reason why the 1^st^ type 1 repeat showed biological activity in our study.

It is of interest that CD148 interacts with the 1^st^, but not the 2^nd^ or 3^rd^, type 1 repeat. Recently, high-resolution structural analysis of the type 1 repeats in TSP1 revealed a significant difference in the linkage between the 1^st^ and 2^nd^ type 1 repeats and the linkage between the 2^nd^ and 3^rd^ type 1 repeats. Between the 1^st^ and 2^nd^ type 1 repeat domains, there is a linker of five residues, which is significantly different from the linker of one residue between the 2^nd^ and 3^rd^ type 1 repeats. In addition, the longer linkage between the 1^st^ and 2^nd^ type 1 repeats is presumed to be very flexible, as distinct from the relatively rigid linkage between the 2^nd^ and 3^rd^ type 1 repeats [45]. These differences in structural features among the type 1 repeats may contribute to the specificity of CD148 binding to the 1^st^ type repeat. Furthermore, another study has shown differences among the type 1 repeats in terms of glycosylation patterns. The first Trp residue in the recognition motif WXXW pattern for C-mannosylation is only modified in the 2^nd^, but not the 1^st^ or 3^rd^, type 1 repeat. The pattern of highly conserved Trp residues at the amino terminal end has been shown only on the 2^nd^ or 3^rd^, but not the 1^st^, type 1 repeat [44]. These differences in the extent of posttranslational covalent modifications may also contribute to the specificity of CD148 binding to the 1^st^ type 1 repeat. In this context, it is of note that TSP2 has an equivalent type 1 repeats domain as TSP1, while other TSP proteins, TSP3, TSP4 and TSP5, lack the procollagen domain and type 1 repeats [23]. Hence, it would be of interest to ask if CD148 also interacts with TSP2 in a similar manner and exhibits biological effects. Further investigation would be required on this subject.

The following issues should be addressed in future studies. First, we successfully created a trimeric TSP1 fragment that activates CD148 more specifically than whole TSP1. Although the TSP1 fragment that contains the procollagen domain alone or a peptide derived from this region showed no anti-angiogenic activity in our and others’ studies [40, 41], Tolsma *et al*. has shown that peptides derived from the procollagen domain inhibits endothelial cell migration in culture and corneal angiogenesis *in vivo* [39]. For this reason, we created the trimeric TSP1 fragment that contains only the 1^st^ type 1 repeat. However, this protein did not work sufficiently due to its instability. Further efforts are required to create a TSP1 agent that is highly specific for CD148, including determination of the CD148-binding TSP1 peptide sequence and creation of a multimeric peptide agent. On the other hand, it was shown that TSP1 is cleaved by ADAMTS1 at the site between the oligomerization sequence and procollagen domain [46]. This cleavage produces the monomeric form of TSP1 protein. Therefore, an effort to generate the uncleavable TSP1 fragment may be required to maximize the efficacy of the TSP1 agent described in this report. Second, a body of literature has shown that CD148 dephosphorylates and suppresses growth factor receptors and their signaling proteins, including EGFR [13, 14], HGFR [5, 15], VEGFR2 [16, 17], ERK1/2 [14, 18], PLCγ1 [19], and p85 [20]. However, these effects were demonstrated by overexpression or knockdown experiments, and the primary signaling events downstream of CD148 are still unclear. Our TSP1 agent would provide an opportunity to address this issue. This study should be a subject of future investigation. In addition, it would be of interest to investigate how CD148 and CD36 pathways interact, cooperate, and induce anti-angiogenesis signals. The trimeric TSP1 fragment that activates both CD148 and CD36 may be a powerful tool for anti-angiogenesis therapy.

In conclusion, the present study characterized the interaction between TSP1 and CD148. Further investigation of this pathway should offer a new strategy and agent for anti-angiogenesis and anti-cancer therapies.

## Supporting Information

S1 Fig Immunoblot analysis of the prepared TSP1 fragments. **(A to C)** TSP1 fragments (0.5 μg) were separated on polyacrylamide gels in reducing or non-reducing conditions and the quality of the proteins was examined by immunoblotting using anti-Myc antibody. Note: No degradation is observed in the prepared TSP1 fragments.
(TIF)

S2 Fig Competition of higher molar excess of TSP1 fragments and AP-TSP1 for binding to CD148-Fc. Protein-A plates conjugated with CD148-Fc (11.3 nM) or equal molar of control Fc were incubated with AP-TSP1 or AP (12 nM) in the presence or absence of TSP1 fragments (100 nM) or whole TSP1 protein (100 nM). The bound AP-TSP1 was assessed by an AP activity assay. The data show mean ± SEM of quadruplicate determinations. Representative data of five independent experiments are shown. ** *P* < 0.05
(TIF)

S3 Fig CD148 knockdown in A431D/CD148wt cells. A431D/CD148wt cells were infected with the lentivirus encoding either CD148-targeting or scrambled shRNA. Cells were lysed in RIPA buffer [50mM Tris pH 8.0, 150 mM NaCl, 1% TritonX-100, 5% sodium deoxycholate, 1% SDS, protease inhibitor cocktail (Roche Life Science)] and cell lysates (50 μg) were subjected to immunoblot analysis with anti-CD148 antibody. Equal loading was evaluated by reprobing the membrane with anti-actin antibody. The ratio of CD148 to actin was measured using ImageJ (NIH) software. Representative data of three independent experiments are shown. Note: CD148-targeting shRNAs reduces CD148 expression by 80–90% in A431D/CD148wt cells.
(TIF)

S4 Fig A monomeric TSP1 fragment containing the procollagen domain and type 1 repeats does not inhibit cell proliferation in A431D/CD148wt cells. A431D/CD148wt cells were treated with the indicated doses of either a trimeric (red triangle) or a monomeric (blue square) TSP1 fragment containing the procollagen domain and type 1 repeats. The effects on cell proliferation were assessed as in Fig 2. Cell density was measured at two days after the addition of protein. The data show mean ± SEM of quadruplicate determinations. Representative data of four independent experiments are shown.
(TIF)

S5 Fig Blocking of AP-TSP1 binding to CD148-Fc by monomeric or trimeric TSP1 fragments. **(A)** Protein-A plates conjugated with CD148-Fc (11.3 nM) or equal molar of control Fc were incubated with AP-TSP1 or AP (12 nM) in the presence or absence of indicated dose of monomeric or trimeric TSP1 fragments as in Fig 1C. The bound AP-TSP1 was assessed by an AP activity assay. The data show mean ± SEM of quadruplicate determinations. Representative data of five independent experiments are shown. **(B)** The results were compared based on the valency of the CD148 binding site in TSP1 fragments, as a monomeric TSP1 fragment has one CD148 binding site (monovalent), while a trimeric fragment has three CD148 binding sites (trivalent).
(TIF)

S6 Fig Immunoblot analysis of A431D/CD36 cells. The expression of CD36 and CD148 was examined in A431D/CD36 cells by immunoblot analysis. Cells were lysed in RIPA buffer [50mM Tris pH 8.0, 150mM NaCl, 1% TritonX-100, 5% sodium deoxycholate, 1% SDS, protease inhibitor cocktail (Roche Life Science)] and 50 μg of cell lysate was subjected to immunoblot analysis with anti-CD148 or anti-CD36 antibodies. Equal loading was evaluated by reprobing the membrane with anti-tubulin antibody. Note: No CD148 expression is observed in A431D/CD36 and A431D cells.
(TIF)

S7 Fig Effects of trimeric and monomeric TSP1 fragments on CD148 catalytic activity, tyrosine phosphorylation of EGFR and ERK1/2, and cellular CD148 distribution. **(A)** A431D/CD148wt cells were treated with vehicle, monomeric (36 nM) or trimeric (12 nM) TSP1 fragments containing the procollagen and 1^st^ type 1 repeats. CD148 catalytic activity (left) and tyrosine phosphorylation of EGFR and ERK1/2 (right) were assessed as in Fig 4. Representative data of four independent experiments is shown. **(B)** A431D/CD148wt cells were starved and treated with monomeric (36 nM) or trimeric (12 nM) TSP1 fragments for 1 h, fixed with 2% paraformaldehyde in PBS for 10 min at RT, then incubated with anti-CD148 antibody (clone 143–41) for 1 h at RT. The immunoreaction was visualized by subsequent incubation with FITC-labeled secondary antibody and photographed using Zeiss LSM 510 META inverted confocal microscopy. Representative data of four independent experiments is shown. Note: CD148 is more accumulated and intensely labeled in cells treated with the trimeric TSP1 fragment. No staining was observed in A431D cells that lack CD148 expression (data not shown).
(TIF)

S8 Fig High dose and longer TSP1 treatment also reduces tyrosine phosphorylation of EGFR and ERK1/2 in A431D/CD148wt cells. **(A)** A431D/CD148wt cells were treated with either vehicle or whole TSP1 protein (214 nM) for 30 min. Tyrosine phosphorylation of EGFR and ERK1/2 was assessed as in Fig 4. Representative data of three independent experiments are shown. (**B)** The expression level of CD148 was examined in A431 cells by immunoblot analysis. Fifty micrograms of cell lysates were subjected to immunoblot analysis with anti-CD148 antibody. Equal loading was evaluated by reprobing the membrane with anti-actin antibody. Note: Relatively low level of CD148 expression in A431 cells.
(TIF)

S9 Fig A trimeric TSP1 fragment containing the procollagen domain and the 1^st^ type 1 repeat reduces tyrosine phosphorylation of VEGFR2 and ERK1/2 in HRMEC cells. HRMEC cells were treated with VEGF (80 ng/ml) with or without a trimeric fragment (12 nM) containing the procollagen domain and the 1^st^ type 1 repeat or whole TSP1 protein (12 nM) for 15 mins. Tyrosine phosphorylation of VEGFR2 and ERK1/2 were investigated as described previously [Proc Natl Acad Sci USA 2012 109(6):1985–90]. Representative data of four independent experiments are shown.
(TIF)

S10 Fig CD148 expression in angiogenic vessels. Gelfoam sponges loaded with 100 ng VEGF were subcutaneously implanted into the dorsal flank of CD148 knockout mice, in which β-galactosidase (LacZ) is expressed under the control of endogenous CD148 promoter. The sponges were rinsed twice with cold PBS and permeabilized with the PBS containing 0.02% NP-40, 0.01% sodium deoxycholate, and 2 mM MgCl_2_ for 30 min at 4°C. Color development was carried out overnight at RT with the PBS containing 0.02% NP-40, 0.01% sodium deoxycholate, 2 mM MgCl_2_, 5 mM potassium ferricyanide, 5 mM potassium ferrocyanide, and 1 mg/ml 5-bromo-4-chloro-3-indolyl-D galactopyranoside (X-gal; Sigma-Aldrich, St. Louis, MO). Paraffin sections were processed and immunostained using anti-vWF antibody and VECTASTATIN Rat IgG ABC Kit (Vector Laboratories, Burlingame, CA). Scale bar, 50 μm. Note: CD148 promoter activity (LacZ) is observed in angiogenic vessels labeled by vWF immunostaining. LacZ staining is also observed in the lumens of blood vessels. This could be hematopoietic cells (or diffusion of LacZ reaction) as CD148 is expressed in hematopoietic populations including macrophages, T cells, and platelets.
(TIF)

S11 Fig A trimeric TSP1 fragment that contains the procollagen domain alone does not inhibit angiogenesis. Gelfoam sponges loaded with vehicle or 100 ng VEGF plus or minus 100 pmol of a trimeric TSP1 fragment containing the procollagen domain were subcutaneously implanted into the dorsal flank of wild-type mice. At day 7, the mice were injected intravenously with 2% TRITC-dextran and vessel density in whole sponges was quantified. TRITC-positive pixel area was measured as in Fig 5B. Data show mean ± SEM of five sponges from independent mice.
(TIF)
