# Supplementary figures and images for: Determination of the CD148-Interacting Region in Thrombospondin-1
Source: PLoS One. 2016 May 5;11(5):e0154916. doi: 10.1371/journal.pone.0154916 (PMC4858292; doi:10.1371/journal.pone.0154916)

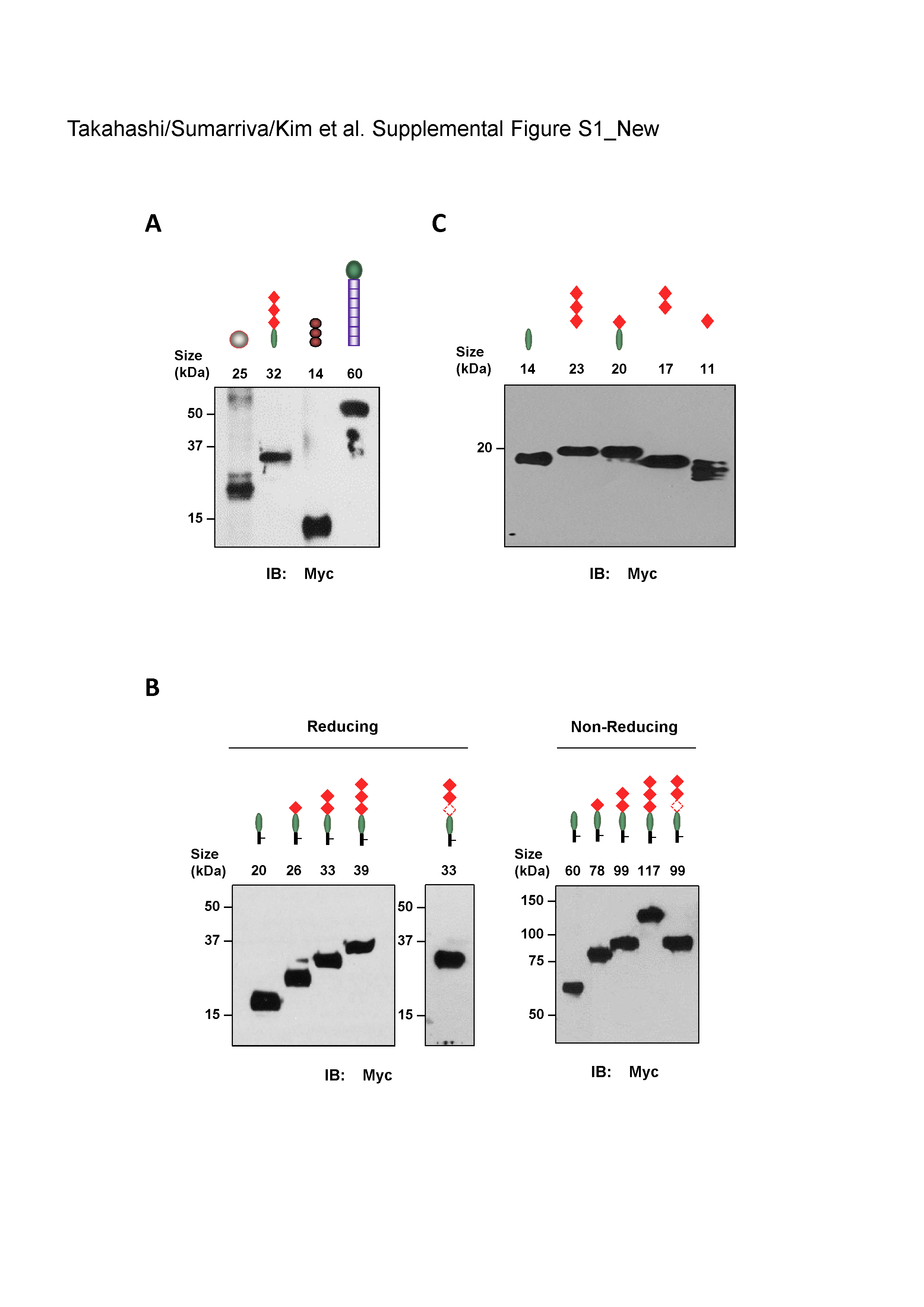

Supplement: S1 Fig — (A to C) TSP1 fragments (0.5 μg) were separated on polyacrylamide gels in reducing or non-reducing conditions and the quality of the proteins was examined by immunoblotting using anti-Myc antibody. Note: No degradation is observed in the prepared TSP1 fragments. (TIF) [file pone.0154916.s001.tif]

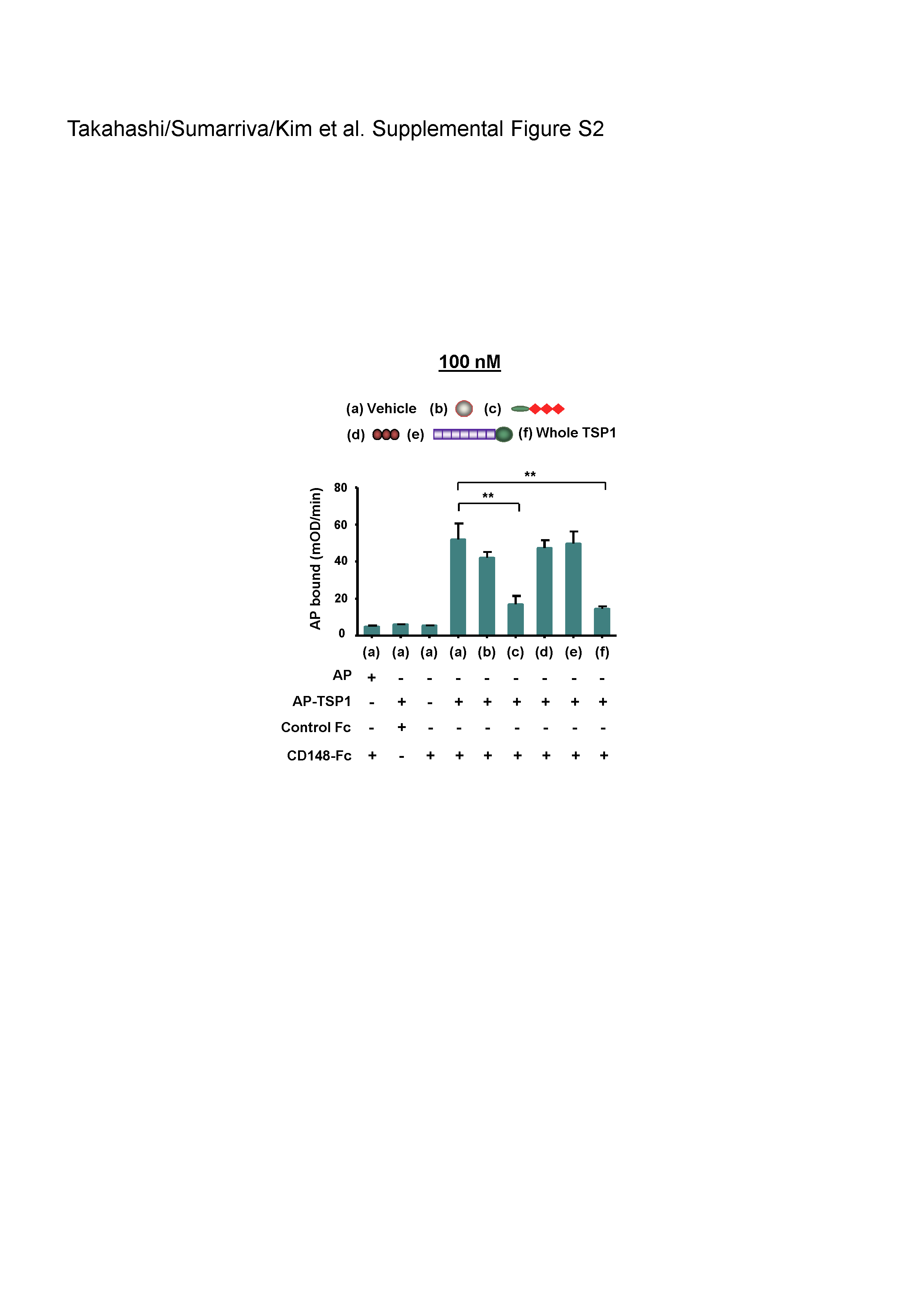

Supplement: S2 Fig — Protein-A plates conjugated with CD148-Fc (11.3 nM) or equal molar of control Fc were incubated with AP-TSP1 or AP (12 nM) in the presence or absence of TSP1 fragments (100 nM) or whole TSP1 protein (100 nM). The bound AP-TSP1 was assessed by an AP activity assay. The data show mean ± SEM of quadruplicate determinations. Representative data of five independent experiments are shown. ** P < 0.05 (TIF) [file pone.0154916.s002.tif]

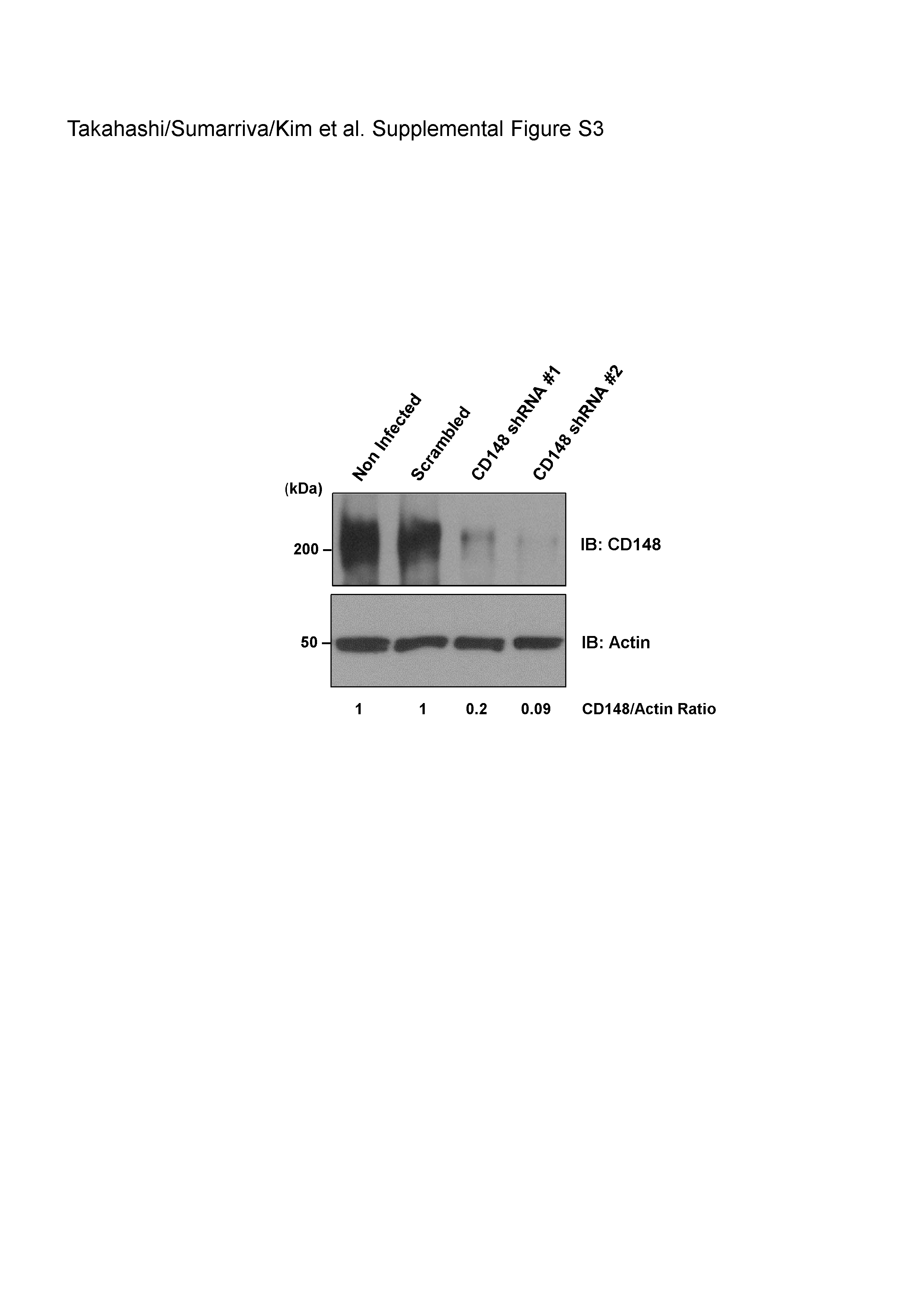

Supplement: S3 Fig — A431D/CD148wt cells were infected with the lentivirus encoding either CD148-targeting or scrambled shRNA. Cells were lysed in RIPA buffer [50mM Tris pH 8.0, 150 mM NaCl, 1% TritonX-100, 5% sodium deoxycholate, 1% SDS, protease inhibitor cocktail (Roche Life Science)] and cell lysates (50 μg) were subjected to immunoblot analysis with anti-CD148 antibody. Equal loading was evaluated by reprobing the membrane with anti-actin antibody. The ratio of CD148 to actin was measured using ImageJ (NIH) software. Representative data of three independent experiments are shown. Note: CD148-targeting shRNAs reduces CD148 expression by 80–90% in A431D/CD148wt cells. (TIF) [file pone.0154916.s003.tif]

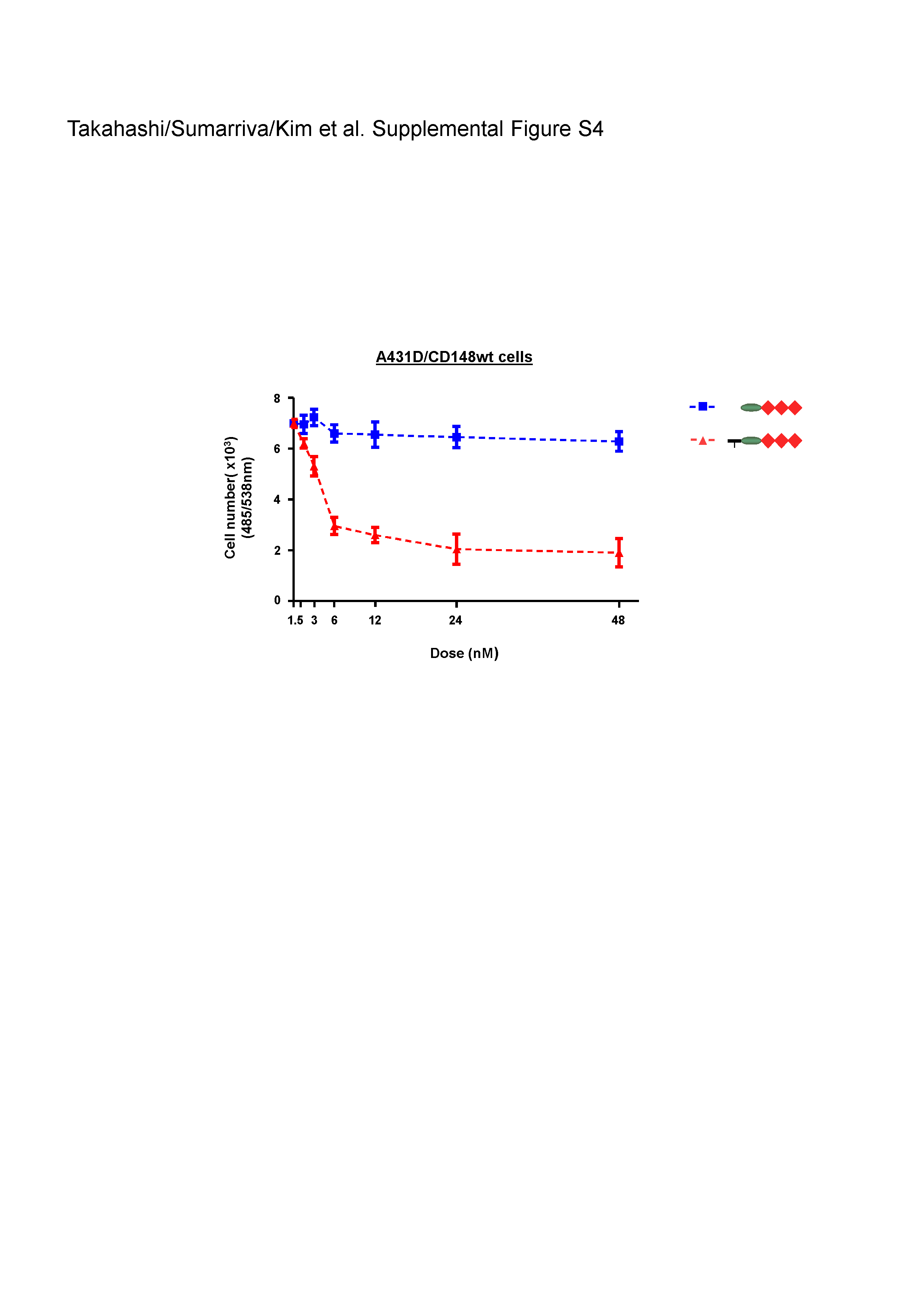

Supplement: S4 Fig — A431D/CD148wt cells were treated with the indicated doses of either a trimeric (red triangle) or a monomeric (blue square) TSP1 fragment containing the procollagen domain and type 1 repeats. The effects on cell proliferation were assessed as in Fig 2. Cell density was measured at two days after the addition of protein. The data show mean ± SEM of quadruplicate determinations. Representative data of four independent experiments are shown. (TIF) [file pone.0154916.s004.tif]

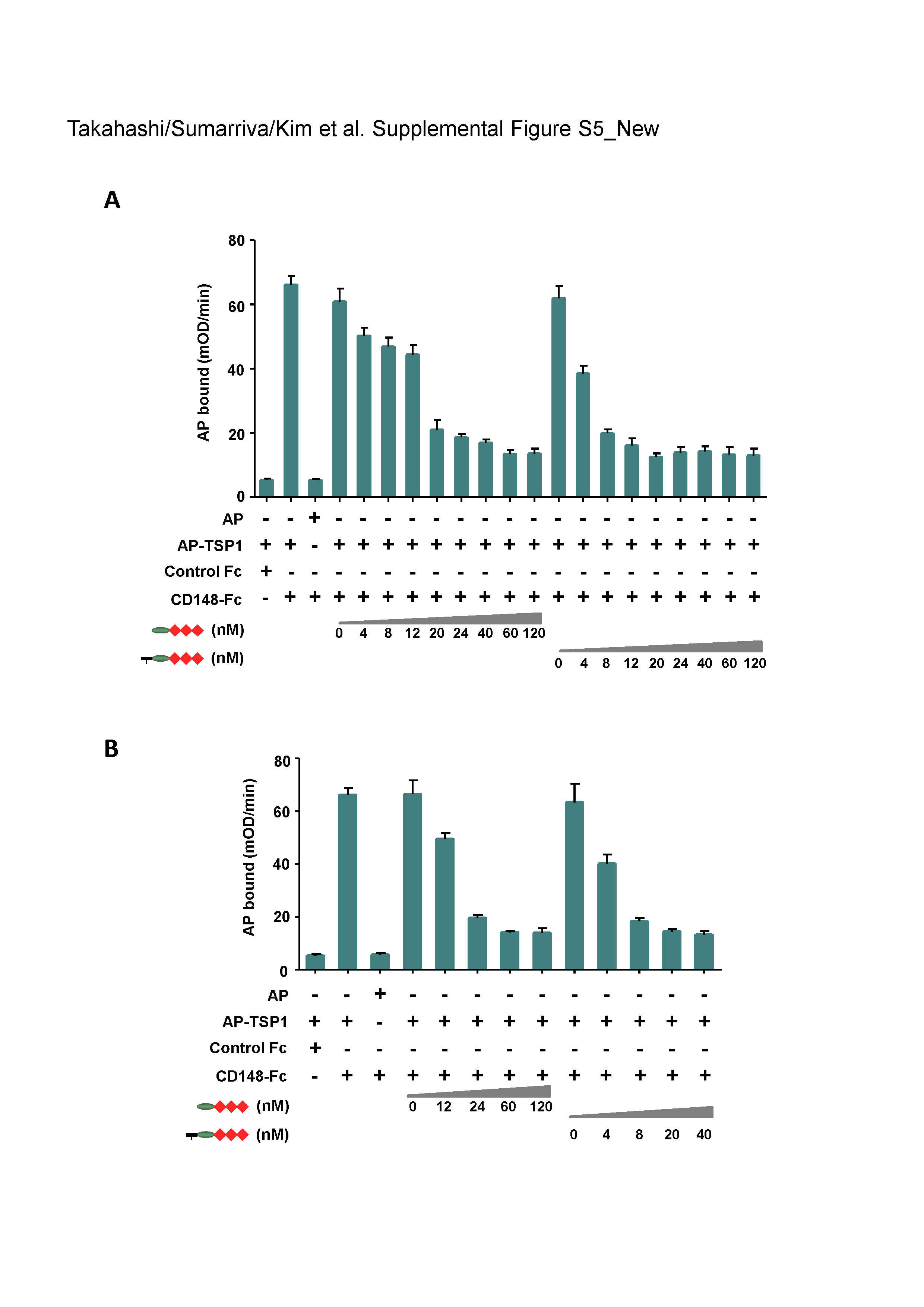

Supplement: S5 Fig — (A) Protein-A plates conjugated with CD148-Fc (11.3 nM) or equal molar of control Fc were incubated with AP-TSP1 or AP (12 nM) in the presence or absence of indicated dose of monomeric or trimeric TSP1 fragments as in Fig 1C. The bound AP-TSP1 was assessed by an AP activity assay. The data show mean ± SEM of quadruplicate determinations. Representative data of five independent experiments are shown. (B) The results were compared based on the valency of the CD148 binding site in TSP1 fragments, as a monomeric TSP1 fragment has one CD148 binding site (monovalent), while a trimeric fragment has three CD148 binding sites (trivalent). (TIF) [file pone.0154916.s005.tif]

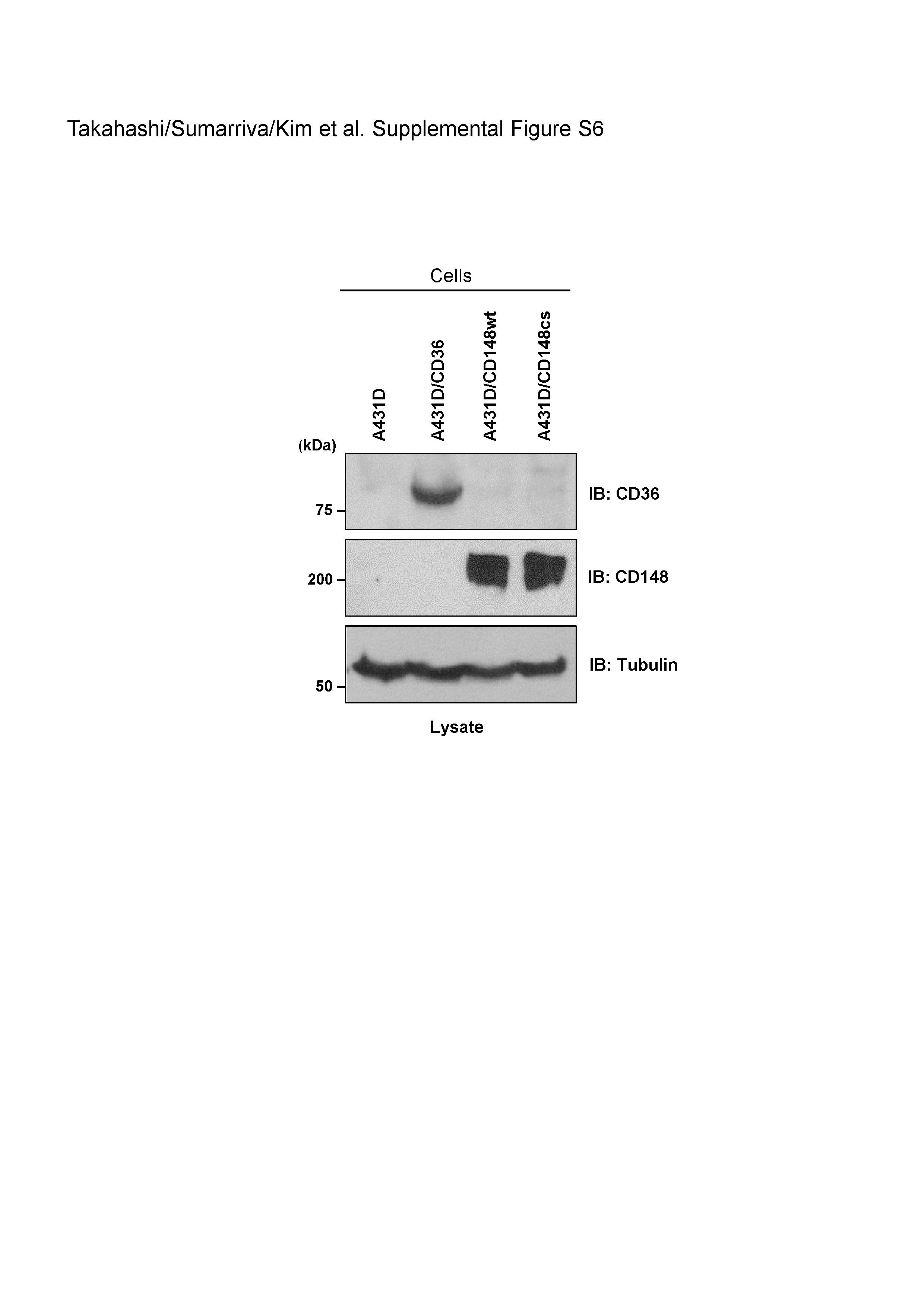

Supplement: S6 Fig — The expression of CD36 and CD148 was examined in A431D/CD36 cells by immunoblot analysis. Cells were lysed in RIPA buffer [50mM Tris pH 8.0, 150mM NaCl, 1% TritonX-100, 5% sodium deoxycholate, 1% SDS, protease inhibitor cocktail (Roche Life Science)] and 50 μg of cell lysate was subjected to immunoblot analysis with anti-CD148 or anti-CD36 antibodies. Equal loading was evaluated by reprobing the membrane with anti-tubulin antibody. Note: No CD148 expression is observed in A431D/CD36 and A431D cells. (TIF) [file pone.0154916.s006.tif]

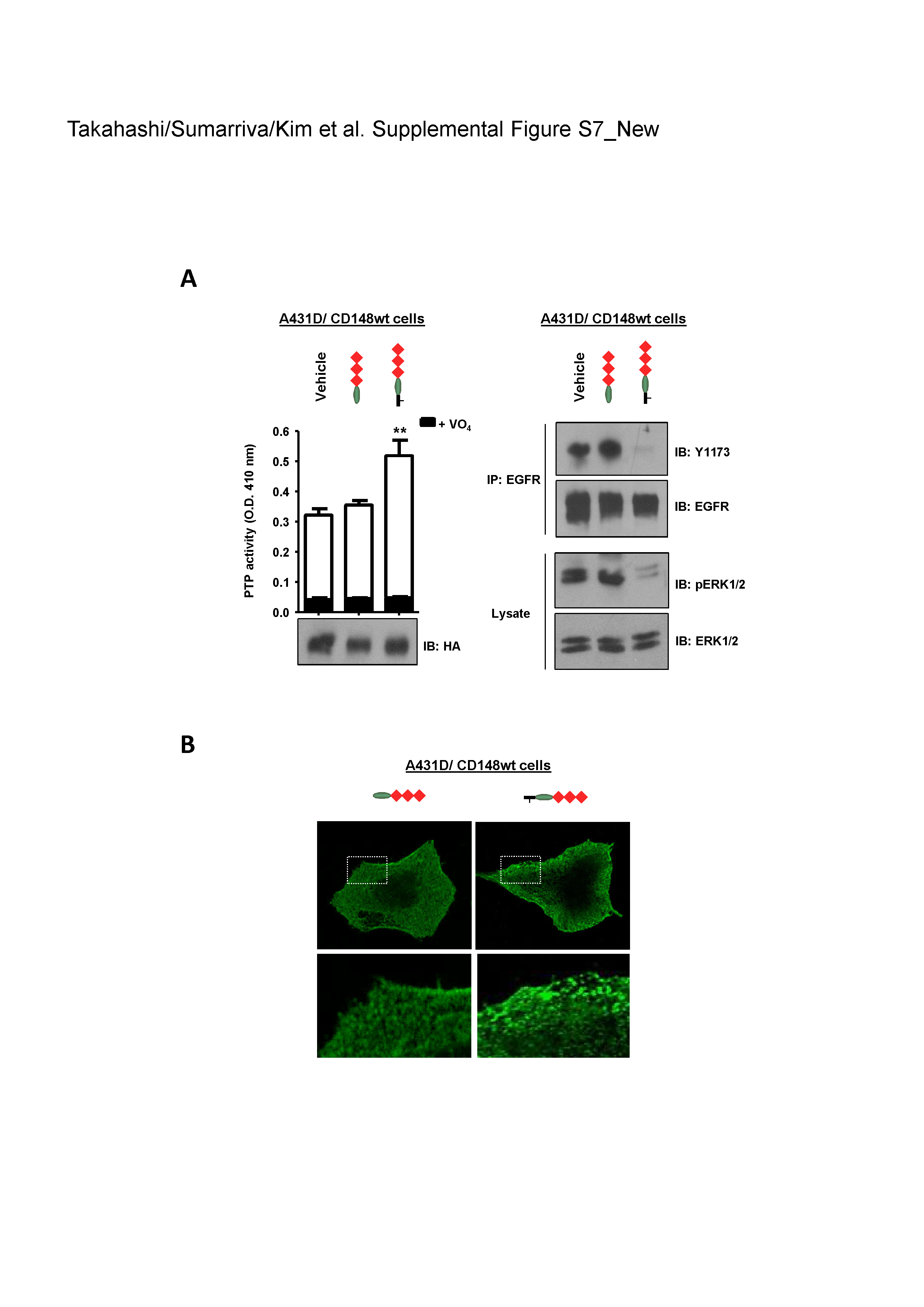

Supplement: S7 Fig — (A) A431D/CD148wt cells were treated with vehicle, monomeric (36 nM) or trimeric (12 nM) TSP1 fragments containing the procollagen and 1st type 1 repeats. CD148 catalytic activity (left) and tyrosine phosphorylation of EGFR and ERK1/2 (right) were assessed as in Fig 4. Representative data of four independent experiments is shown. (B) A431D/CD148wt cells were starved and treated with monomeric (36 nM) or trimeric (12 nM) TSP1 fragments for 1 h, fixed with 2% paraformaldehyde in PBS for 10 min at RT, then incubated with anti-CD148 antibody (clone 143–41) for 1 h at RT. The immunoreaction was visualized by subsequent incubation with FITC-labeled secondary antibody and photographed using Zeiss LSM 510 META inverted confocal microscopy. Representative data of four independent experiments is shown. Note: CD148 is more accumulated and intensely labeled in cells treated with the trimeric TSP1 fragment. No staining was observed in A431D cells that lack CD148 expression (data not shown). (TIF) [file pone.0154916.s007.tif]

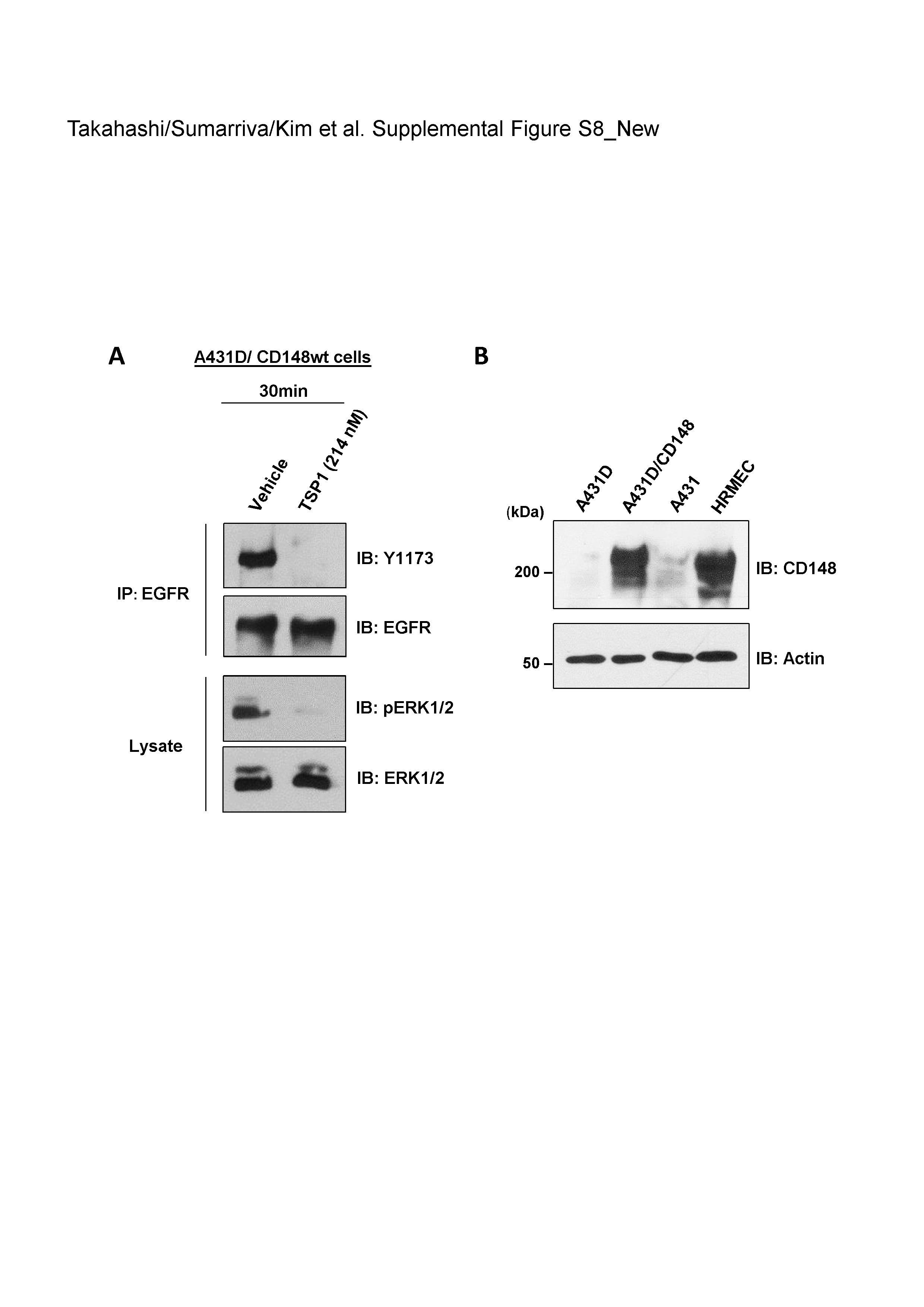

Supplement: S8 Fig — (A) A431D/CD148wt cells were treated with either vehicle or whole TSP1 protein (214 nM) for 30 min. Tyrosine phosphorylation of EGFR and ERK1/2 was assessed as in Fig 4. Representative data of three independent experiments are shown. (B) The expression level of CD148 was examined in A431 cells by immunoblot analysis. Fifty micrograms of cell lysates were subjected to immunoblot analysis with anti-CD148 antibody. Equal loading was evaluated by reprobing the membrane with anti-actin antibody. Note: Relatively low level of CD148 expression in A431 cells. (TIF) [file pone.0154916.s008.tif]

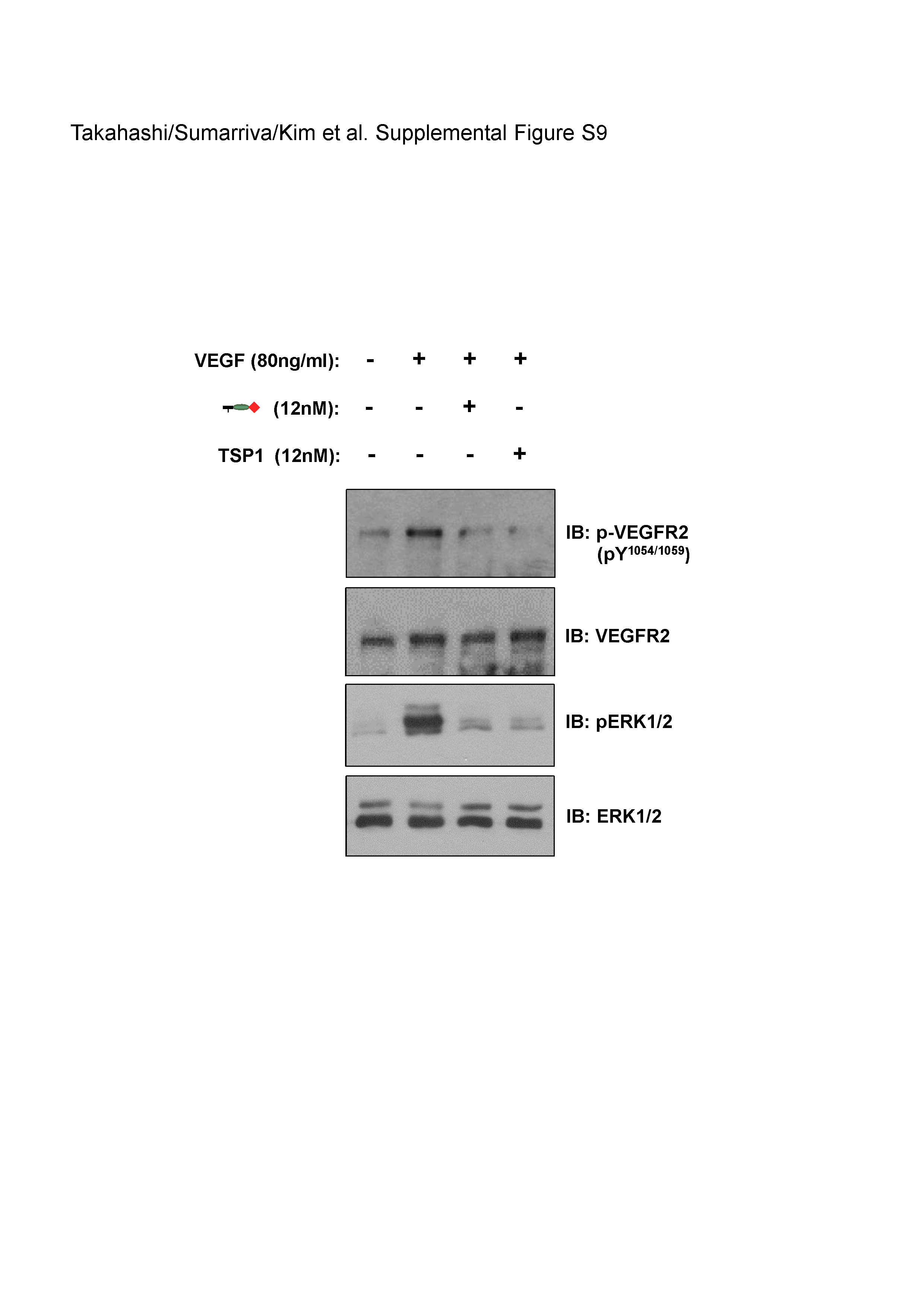

Supplement: S9 Fig — HRMEC cells were treated with VEGF (80 ng/ml) with or without a trimeric fragment (12 nM) containing the procollagen domain and the 1st type 1 repeat or whole TSP1 protein (12 nM) for 15 mins. Tyrosine phosphorylation of VEGFR2 and ERK1/2 were investigated as described previously [Proc Natl Acad Sci USA 2012 109(6):1985–90]. Representative data of four independent experiments are shown. (TIF) [file pone.0154916.s009.tif]

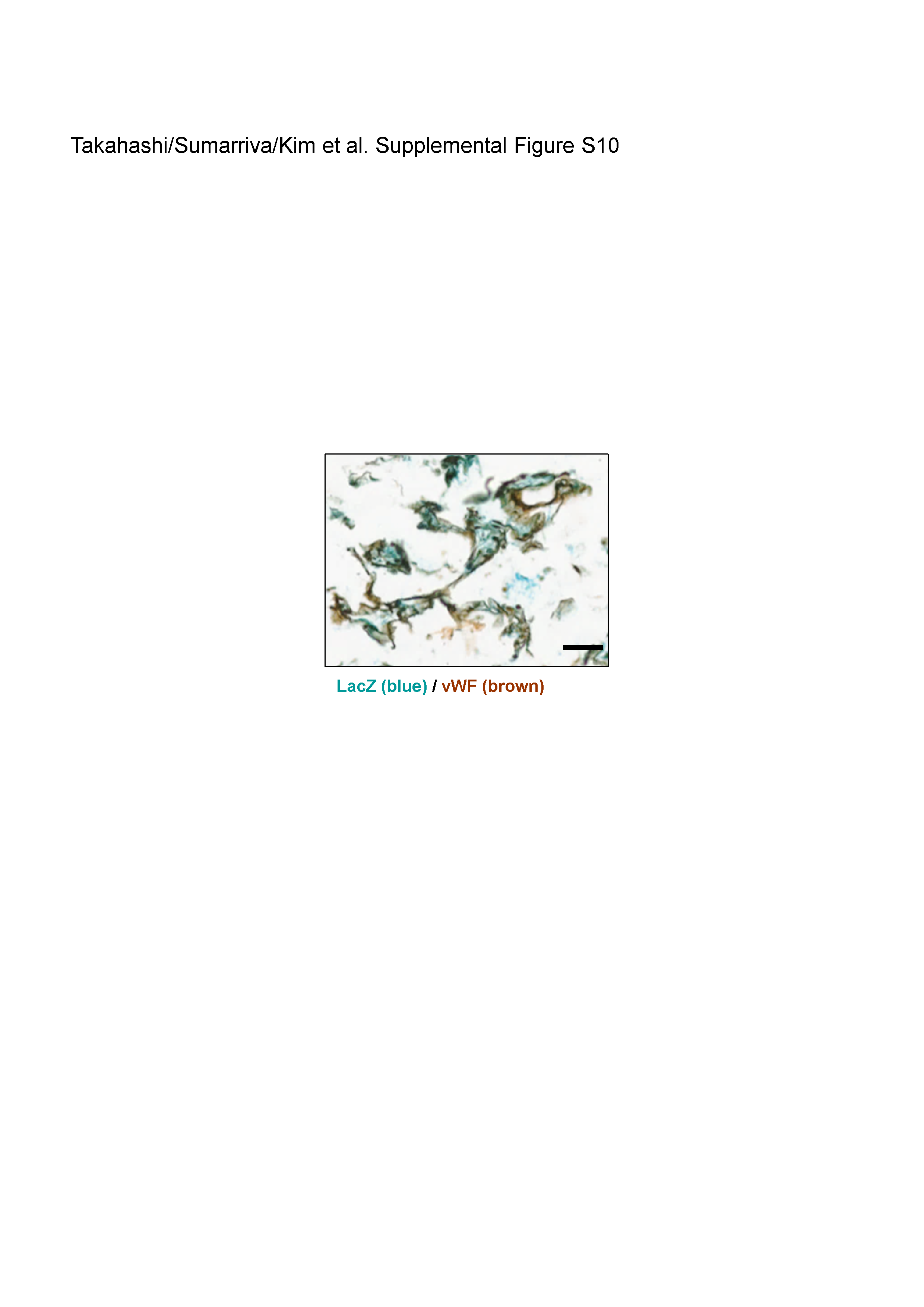

Supplement: S10 Fig — Gelfoam sponges loaded with 100 ng VEGF were subcutaneously implanted into the dorsal flank of CD148 knockout mice, in which β-galactosidase (LacZ) is expressed under the control of endogenous CD148 promoter. The sponges were rinsed twice with cold PBS and permeabilized with the PBS containing 0.02% NP-40, 0.01% sodium deoxycholate, and 2 mM MgCl2 for 30 min at 4°C. Color development was carried out overnight at RT with the PBS containing 0.02% NP-40, 0.01% sodium deoxycholate, 2 mM MgCl2, 5 mM potassium ferricyanide, 5 mM potassium ferrocyanide, and 1 mg/ml 5-bromo-4-chloro-3-indolyl-D galactopyranoside (X-gal; Sigma-Aldrich, St. Louis, MO). Paraffin sections were processed and immunostained using anti-vWF antibody and VECTASTATIN Rat IgG ABC Kit (Vector Laboratories, Burlingame, CA). Scale bar, 50 μm. Note: CD148 promoter activity (LacZ) is observed in angiogenic vessels labeled by vWF immunostaining. LacZ staining is also observed in the lumens of blood vessels. This could be hematopoietic cells (or diffusion of LacZ reaction) as CD148 is expressed in hematopoietic populations including macrophages, T cells, and platelets. (TIF) [file pone.0154916.s010.tif]

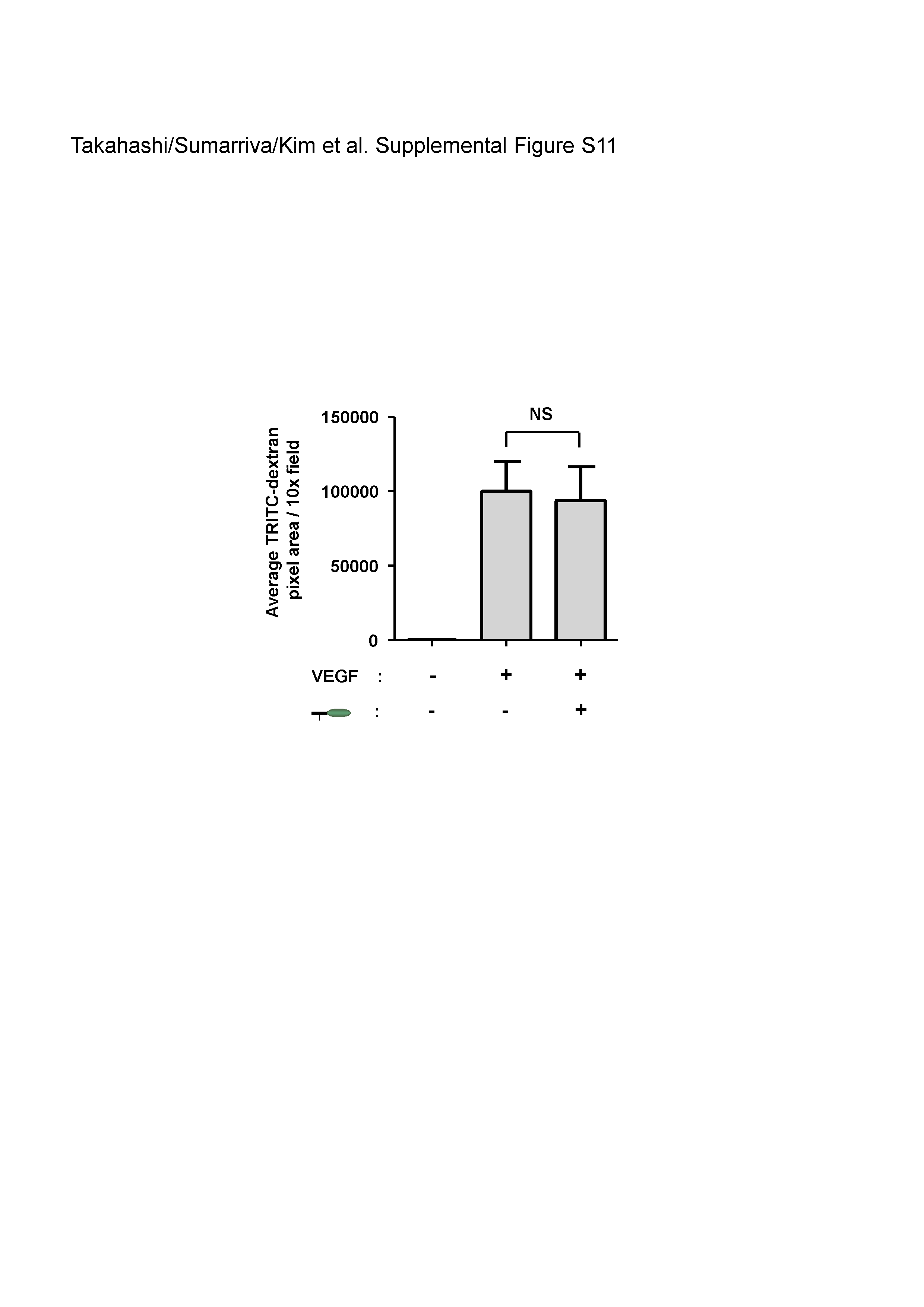

Supplement: S11 Fig — Gelfoam sponges loaded with vehicle or 100 ng VEGF plus or minus 100 pmol of a trimeric TSP1 fragment containing the procollagen domain were subcutaneously implanted into the dorsal flank of wild-type mice. At day 7, the mice were injected intravenously with 2% TRITC-dextran and vessel density in whole sponges was quantified. TRITC-positive pixel area was measured as in Fig 5B. Data show mean ± SEM of five sponges from independent mice. (TIF) [file pone.0154916.s011.tif]
